# *Colobopsis explodens* sp. n., model species for studies on “exploding ants” (Hymenoptera, Formicidae), with biological notes and first illustrations of males of the *Colobopsis cylindrica* group

**DOI:** 10.3897/zookeys.751.22661

**Published:** 2018-04-19

**Authors:** Alice Laciny, Herbert Zettel, Alexey Kopchinskiy, Carina Pretzer, Anna Pal, Kamariah Abu Salim, Mohammad Javad Rahimi, Michaela Hoenigsberger, Linda Lim, Weeyawat Jaitrong, Irina S. Druzhinina

**Affiliations:** 1 2nd Zoological Department, Natural History Museum Vienna, Burgring 7, 1010 Vienna, Austria; 2 Department of Theoretical Biology, Althanstraße 14, 1090 Vienna, Austria; 3 Research Area Biochemical Technology, Institute of Chemical, Environmental and Biological Engineering, TU Wien, Gumpendorfer Straße 1a, 1060 Vienna, Austria; 4 Environmental and Life Sciences, Universiti Brunei Darussalam, Jalan Tungku Link, Bandar Seri Begawan BE 1410, Brunei Darussalam; 5 Center for Analytical Chemistry, Department of Agrobiotechnology, University of Natural Resources and Life Sciences, Vienna (BOKU), Konrad-Lorenz-Straße 20, 3430 Tulln, Austria; 6 Chemical Sciences, Universiti Brunei Darussalam, Jalan Tungku Link, Bandar Seri Begawan, BE 1410, Brunei Darussalam; 7 Thailand Natural History Museum, National Science Museum, Technopolis, Khlong 5, Khlong Luang, Pathum Thani, 12120 Thailand

**Keywords:** autothysis, behavioural ecology, Camponotini, *Colobopsis*, Formicidae, integrative taxonomy, male morphology, molecular biology, morphometry, new species, new status, new synonymy, phylogeny, Southeast Asia, taxonomy

## Abstract

A taxonomic description of all castes of *Colobopsis explodens* Laciny & Zettel, **sp. n.** from Borneo, Thailand, and Malaysia is provided, which serves as a model species for biological studies on “exploding ants” in Southeast Asia. The new species is a member of the *Colobopsis cylindrica* (COCY) group and falls into a species complex that has been repeatedly summarized under the name *Colobopsis saundersi* (Emery, 1889) (formerly *Camponotus saundersi*). The COCY species group is known under its vernacular name “exploding ants” for a unique behaviour: during territorial combat, workers of some species sacrifice themselves by rupturing their gaster and releasing sticky and irritant contents of their hypertrophied mandibular gland reservoirs to kill or repel rivals. This study includes first illustrations and morphometric characterizations of males of the COCY group: *Colobopsis explodens* Laciny & Zettel, **sp. n.** and *Colobopsis badia* (Smith, 1857). Characters of male genitalia and external morphology are compared with other selected taxa of Camponotini. Preliminary notes on the biology of *C. explodens* Laciny & Zettel, **sp. n.** are provided. To fix the species identity of the closely related *C. badia*, a lectotype from Singapore is designated. The following taxonomic changes within the *C. saundersi* complex are proposed: *Colobopsis solenobia* (Menozzi, 1926), **syn. n.** and *Colobopsis trieterica* (Menozzi, 1926), **syn. n.** are synonymized with *Colobopsis corallina* Roger, 1863, a common endemic species of the Philippines. *Colobopsis saginata* Stitz, 1925, **stat. n**., hitherto a subspecies of *C. badia*, is raised to species level.

## Introduction

The *Colobopsis cylindrica* (COCY) group likely represents a monophyletic clade containing Southeast Asian ant species with distinctive hypertrophied mandibular gland reservoirs. In territorial combat, minor workers use the sticky and irritant contents of their enlarged mandibular gland reservoirs to kill or repel rival arthropods. In species where this defensive behaviour is more advanced, this happens via the characteristic suicidal “exploding” by voluntary rupture of the gastral integument (autothysis) (Cook 2008). This behaviour was first mentioned by Viehmeyer as early as 1916, and subsequently described in detail by Maschwitz and Maschwitz (1974), as well as Davidson et al. (2012), and Shorter and Rueppell (2012).

The Bornean members of the COCY group have been the subject of various ecological (e.g., Cook 2008, Davidson et al. 2007, 2009, 2016), morphological (Davidson et al. 2012, Laciny et al. 2017) and chemical (Jones et al. 2004, Hoenigsberger et al. in prep.) studies in the past. Based on the results of previous investigations, in 2014 an interdisciplinary research project started to explore the evolution and ecological significance of autothysis in the COCY group. From the surroundings of the Kuala Belalong Field Studies Centre (KBFSC) in Brunei, at least 15 species are known (Davidson et al. 2007), most of which are probably new to science. One species, previously referred to as “yellow goo” (Davidson et al. 2007) or “YG” (Davidson et al. 2016) for the bright yellow colour of its mandibular gland secretion, was found to have a large colony just at the KBFSC. As this abundant species frequently exhibits characteristic autothysis behaviour and can be observed *in situ* and *in vitro*, it became the main object of behavioural and chemical experiments, and a model species for biological studies on “exploding ants”. Preliminary taxonomic and molecular analyses revealed that this morphospecies is in fact an undescribed species. As the revision of the COCY group is still ongoing (I. Druzhinina et al. in prep.), the aim of this paper is to provide a valid name, *Colobopsis explodens* Laciny & Zettel, sp. n., for subsequent use in the various behavioural, chemical, microbiological, and evolutionary publications currently in preparation. Within this study, we employ the multidisciplinary concept of integrative taxonomy (*sensu* Schlick-Steiner et al. 2010) by combining morphometric, ecological, and molecular data. We provide a taxonomic description of all castes of *Colobopsis explodens* sp. n. including males. Illustrations and morphometric characterizations of males of the COCY group had not been previously published. We compare males of *Colobopsis explodens* sp. n. with the newly illustrated male of *C. badia* (Smith, 1857) to highlight species-specific characters in the complex. Morphological characters of the male, including genitalia, are also compared with other selected taxa of Camponotini. Based on field observations, the first records on the natural history and biology of *Colobopsis explodens* sp. n. are provided.

## Materials and methods

### Sampling-sites and imaging of living ants

The primary field research took place in the lowland dipterocarp rainforest at the Kuala Belalong Field Studies Centre (KBFSC), Temburong District, Brunei Darussalam (4°32'48.2"N, 115°09'27.9"E), where *Colobopsis explodens* sp. n. was sampled during five collecting trips (each of 30 days duration) encompassing different seasons from 2014 to 2016.

The behaviour of *C. explodens* sp. n. was observed at multiple nesting sites on several height-levels, starting from the forest floor and understory up to the canopy and emergent layer. The activity of ants was recorded *in situ* and *in vitro* using a CANON 70D Digital SLR Camera with a CANON EF 100 mm macro lens and a Tamron AF 28–200 mm F/3.8–5.6 XR Di aspherical (IF) macro zoom lens (Suppl. material 2: S2a). For macro and close-up filming the Neewer adjustable LED light with LCD display was used. When necessary, the camera was mounted with the use of a Manfrotto Gorillapod 494RC2 tripod. The movie (Suppl. material 7) was annotated and cut using Corel VideoStudio X10 Software.

Sampling of *Colobopsis badia* in southern Thailand was conducted by H. Zettel and W. Jaitrong in June 2016. The sampling site was located in the Khao Chong Botanical Garden, near the Ton Pliw Waterfall (07°32'34"N, 99°47'33"E); a single male specimen was caught at a light at the Botanical Garden headquarters.

### Host trees and activity assessment

Nesting habits of *C. explodens* sp. n. were observed based on the model colony occupying several trees and an artificial nest (nest #38, Fig. 9) in direct vicinity to the kitchen facility at KBFSC. The artificial nest consisted of a 100 cm tall and 6 cm wide square wooden stake, with a cavity of approximately 15 mm in diameter drilled into the centre and a 4 mm wide entrance hole in the top third of the stake. The nest was painted with green acrylic paint and fastened to a small tree with rope (for detailed method of construction, see Davidson et al. 2009 and Laciny et al. 2017). The host trees were identified by comparison with type samples preserved in the herbarium of Universiti Brunei Darussalam, Brunei. The main host tree was DNA barcoded (see Suppl. material 6 “accession numbers”).

The activity of *C. explodens* sp. n. occupying artificial nest #38 was observed from 14^th^ to 30^th^ November of 2015 at different times during the day, for 30 minutes each by counting the ants entering and leaving the nest. Temperature, barometric pressure, and weather conditions were recorded, as well as any observed noteworthy behaviour (see Fig. 9; Suppl. material 6 “activity”).

### DNA Extraction, PCR amplification, and Sanger sequencing

DNA extraction, gene fragment amplification, and sequencing were performed for minor worker ants of five different taxa (*C. explodens* sp. n., *C. badia*, C. nr. saundersi, *C. aruensis* Karawajew, 1933, and *C. cylindrica* (Fabricius, 1798)), as well as for mandibular gland reservoir content of *C. explodens* sp. n., one symbiotic cricket (*Camponophilus* sp.) from artificial nest #38, and the host plant of *C. explodens* sp. n. (*Shorea johorensis*).

For DNA barcoding of ant specimens, DNA was extracted from ant legs using Qiagen’s tissue QIAamp DNA Micro kit following the manufacturer’s protocol (Qiagen, Venlo, Netherlands). To obtain sufficient DNA quantity for further processing, the amount of legs used per sample varied. For the DNA extraction of queens, a minimum of three legs of one individual were transferred into one 1.5 ml microcentrifuge tube and frozen with liquid nitrogen. Three legs of one individual was also the minimum amount for males and major workers. For minor workers, all legs from two to four individuals were pooled (see Suppl. material 6 “accession numbers”). The frozen legs were ground into small pieces with disposable pestles attached to a pestle motor (Kimble, Vineland, NJ, USA). Subsequent steps were performed according to manufacturer's instructions with the following exceptions: sample lysis for 20 hours, final elution step with 25–50 µl elution buffer. To assess the purity of the extraction, DNA concentration and 260/280 nm ratio were measured with a NanoDrop ND-1000 Spectrophotometer (Software Version ND-1000 v.3.8.1, Thermo Fisher Scientific, MA, USA).

For DNA barcoding of symbiotic crickets, DNA was extracted from one whole specimen applying the same procedure as used for ants legs, but with a pretreatment with an enzymatic lysis buffer (Tris·Cl 20 mM, pH 8.0, sodium EDTA 2 mM, Triton X-100 1.2%, add lysozyme to 20 mg/ml for 60 min).

For DNA barcoding of the host plant, 100 mg of a leaf were ground with mortar and pestle under the use of liquid nitrogen and DNA was extracted using Qiagen’s DNeasy Plant Mini Kit according to manufacturer’s instructions.

For the ants, the gene fragments cytochrome C oxidase subunit I and II (COI, COII), cytochrome B (cytB), and carbamoyl-phosphate synthase II (cad) were amplified, for the cricket only COI. Additionally, a fragment of 16S rRNA was amplified from the DNA extracted from the mandibular gland content of *C. explodens* sp. n. minor workers, to assess the presence of bacteria. For the plant, the gene fragment maturase K (matK) was amplified. Primer sequences and specific annealing temperatures are given in Tab. 1. Final concentrations for PCR were 1× GoTaq Flexi Buffer (Promega, Madison, Wisconsin, USA), 0.16 mM dNTP’s, 3 mM MgCl_2_ (Promega), 0.4 µM forward and reverse primer (Microsynth, Balgach, Switzerland), 0.8 Units GoTaqG2 Flexi polymerase (Promega) and 2–50 ng sample (diluted with HPLC water, ROTH), in a final volume of 50 µl. PCR was performed with a Biometra T3 Thermocycler (Biometra, Göttingen, Germany) with the following conditions: 2 min at 94 °C, 35 cycles of 1 min at 94 °C, 1 min at primer specific annealing temperature, 90 sec at 72 °C and finally 7 min at 72 °C. PCR products were separated by 1.5% agarose gel electrophoresis. PCR products were purified using mi-PCR Purification Kit (Metabion, Planegg, Germany) and one direction sequencing was performed at Microsynth (Austria).

Sequences are deposited in NCBI GenBank. Accession numbers for ant specimens are given in Table 2; see Suppl. material 6 “accession numbers” for additional details. Sequences of non-ant material are deposited under MG582639 for COI of myrmecophilous crickets (*Camponophilus* sp.), MF993320 for matK of *Shorea johorensis* and MF996752 for 16S rRNA of cf. *Blochmannia* (Enterobacteriales).

**Table 1.** Primers used in this study.

Gene	Name	Sequence 5’–3’	Length [bp]	GC content [%]	Fragment Length [bp]	Annealing Temp [°C]	Reference
COI	LCO1490-F	GGTCAACAAATCATAAAGATATTGG	25	32	709	45	Chen et al. 2013
	HCO2198-R	TAAACTTCAGGGTGACCAAAAAATCA	26	35			
COII	J2791-F	ATACCHCGDCGATAYTCAGA	20	40–55	858	51	Chen et al. 2013
	H3665-R	CCACARATTTCWGAACATTG	20	35–40			
cytB	CB11400-F	TATGTACTACCHTGAGGDCAAATATC	26	35–42	485	45	Chen et al. 2013
	CB11884-R	ATTACACCNCCTAATTTATTAGGRAT	26	27–35			
cad	CD1423EF	AGGTRATACRATCGGARAGRCCDGA	25	40–60	800	55	Ward et al. 2010
	CD1910R	CCGAGRGGRTCRACRTTYTCCATRTTRCAYAC	32	38–63			
matK	472F	CCCRTYCATCTGGAAATCTTGGTTC	25	44–52	750	47	Yu et al. 2011
	1248R	GCTRTRATAATGAGAAAGATTTCTGC	26	31–38			
16S rRNA	fD1	AGAGTTTGATCCTGGCTCAG	20	50	1500	56	Weisburg et al. 1991
	rP1	ACGGTTACCTTGTTACGACTT	21	43			

**Table 2.** List of sequence accession numbers in NCBI GenBank. * Nucleotide sequences from NCBI GenBank.

TUCIM	Other IDs	Organism	ng/µl	COI	COII	cytB	cad
5053		*C. explodens* sp. n.	14.7	MF993252	MF993269	MF993286	MF993304
5056		*C. explodens* sp. n.	19.7	MF993253	MF993270	MF993287	MF993305
5080		*C. explodens* sp. n.	10.4	MF993254	MF993271	MF993288	MF993306
5098		*C. explodens* sp. n.	8.6	MF993256	MF993273	MF993290	MF993308
5104		*C. explodens* sp. n.	16.6	MF993257	MF993274	MF993291	MF993309
5148		*C. explodens* sp. n.	6	MF993258	MF993275	MF993292	MF993310
5185		*C. explodens* sp. n.	7.3	MF993259	MF993276	MF993293	–
5205		*C. explodens* sp. n.	29.8	MF993260	MF993277	MF993294	MF993311
6600		*C. explodens* sp. n.	8	–	MF993284	–	–
5855		*C. explodens* sp. n.	16.9	MF993262	MF993278	MF993297	MF993314
5856		*C. explodens* sp. n.	28.2	MF993263	MF993279	MF993298	MF993315
5942		*C. explodens* sp. n.	34.3	MF993264	MF993280	MF993299	MF993316
5943		*C. explodens* sp. n.	142.1	MF993265	MF993281	MF993300	–
	YG*	*C. explodens* sp. n.	n.a.	EF634201		–	–
6461		*C. badia*	21.3	MF993266	MF993282	MF993301	MF993317
6463		*C. badia*	5.4	MF993267	MF993283	MF993302	MF993318
6601		*C. badia*	17.91	MF993268	MF993285	MF993303	MF993319
5698		C. nr. saundersi	50.1	KU975365.1	KU975366.1	MF993296	MF993313
	CH*	C. cf. cylindrica	n.a.	EF634198		–	–
5086		*C. cylindrica*	26	MF993255	MF993272	MF993289	MF993307
5300	CAMP004	*C. aruensis*	169.1	MF993261	–	MF993295	MF993312
	Cflor36*	*Camponotus floridanus*	n.a.	AY334397	–	–	–

### Phylogenetic analysis

GapStreeze v. 2.1.0 (https://www.hiv.lanl.gov/content/sequence/GAPSTREEZE/gap.html) was used for COI gene alignment with 95 % gap tolerance in order to retain only the conserved region. The individual gene alignments were subjected to best substitution model selection using the BIC criterion in SMS (Lefort et al. 2017). Consecutively, HKY85, HKY85+I, HKY85+G, and GTR+G were chosen as best substitution models for genes cad, cytB, COI, and COII respectively. The concatenated alignment was partitioned for each locus using MrBayes v. 3.2.5 (Ronquist et al. 2012), and the respective substitution models were assigned to each partition. The substitution and branch length estimates were allowed to vary independently between each partition. Priors for an exponential distribution with mean 1 to all branch lengths and to all shape parameters were assigned for all four partitions. Metropolis-coupled Markov chain Monte Carlo (MCMCMC) sampling was performed using MrBayes v. 3.0B4 (Ronquist et al. 2012) with two simultaneous runs of four incrementally heated chains that performed 1 million generations. Bayesian posterior probabilities (PP) were obtained from the 50 % majority rule consensus of trees sampled every 100 generations after removing the first 25 % of trees using the “burnin” command. According to the protocol of Leache and Reeder (2002), PP values higher than 0.94 were considered significant. The phylogenetic trees were visualized in FigTree v. 1.4.3 (Rambaut 2016) and then annotated using vector graphic software.

### Morphological methods

All specimens used for morphometry were card-mounted, individually numbered, and measured at magnifications from 25.6× up to 256× with a Nikon SMZ1500 binocular microscope. Genital structures of two male specimens were dissected and mounted separately. Results represent minimum and maximum values for each morph; in cases where a character could not be measured in all individuals, the number of measured specimens is given in parentheses. The complete dataset of measurements is provided in Suppl. material 6 “measurements”.

Measurements and indices (* = only gynes and males)

**EL** Eye length. Maximum diameter of compound eye, measured in lateral view.

**FeL** Femur length. Maximum length of metafemur, measured from base to apex.

FWL* Forewing length. Length of forewing, measured from tegula to distal tip.

**HaL** Hair length. Length of the longest standing hair on first gastral tergite, measured from base to apex.

**HL** Head length. Maximum length of head in full-face view, excluding mandibles, measured from anteriormost point of clypeus to posterior-most point of head vertex, parallel to midline.

**HS** Head size. (HW + HL) / 2.

**HW** Head width. Maximum width of head in full-face view (including eyes if protruding; only in gynes).

**ML** Mesosoma length. Measured laterally from anterior surface of pronotum proper (excluding collar) to posterior extension of propodeal lobes.

MSW* Mesoscutum width. Maximum diameter of mesoscutum, measured dorsally.

**NH** Node height. Height of petiolar node, measured laterally, from the intersection point of the axes of maximum height and length to dorsal apex

OcD* Ocellar distance. Minimum distance between lateral ocelli, measured between median borders.

OcW* Ocellus width. Maximum diameter of median ocellus.

OED* Ocellar eye distance. Minimum distance between lateral ocellus and outer border of compound eye.

**PH** Petiole height. Maximum height of petiole in lateral view, measured from ventral-most point of petiolar sternum to dorsal apex.

**PL** Petiole length. Maximum length of petiole in lateral view, measured from inflexion point of anterior constriction to posterior margin, perpendicular to axis of maximum height.

**PS5** Length of maxillary palp segment V, measured from base to apex.

**PS6** Length of maxillary palp segment VI, measured from base to apex.

**SL** Scape length. Maximum length of antennal scape in dorsal view excluding basal neck and condyle.

**SW** Scape width. Maximum width of antennal scape, measured dorsally.

**TL** Total length. The added lengths of head (excluding mandibles), mesosoma, petiole, and gaster.

2r* Maximum length of 2^nd^ radial crossvein (see Figs 5e, 6b).

4Rs+M* Length of 4^th^ radial sector fused with median (see Figs 5e, 6b).

**CI** Cephalic index. HW / HL × 100

**EI** Eye Index. EL / HW × 100

**FeI** Femur Index. FeL / HW × 100

OI* Ocellar Index: OED / OcD × 100

**PI** Petiole Index. PH / PL × 100

**PSI** Palp Segment Index. (PS5 + PS6) / HS × 100

**SI** Scape index. SL / HW × 100

**SWI** Scape width index. SW / SL × 100

WVI* Wing Vein Index. 4RsM / 2r × 100

Digital stacked images of most specimens (Figs 2–6) were acquired with a Leica DFC camera attached to a Leica MZ16 binocular microscope with Leica Application Suite v3 and stacked with Zerene-Stacker 64-bit. Images of labels were taken with a Nikon D60 camera with an AF-S Micro Nikkor 105 mm objective and an EM-140 DG macro ring flash. Photographs of genital structures of males (Figs 7, 10c–f) as well as of the male *C. badia* specimen (Fig. 10 a, b) were created with the help of Leica Application Suite v3.8, using a Leica DFC450 camera attached to a Leica Z16APO optics carrier. All images were processed with Adobe Photoshop 7.0.

### Material examined

Type material of *C. explodens*: **Holotype** (minor worker): Brunei, Temburong, Kuala Belalong Field Studies Centre, 04°33'N, 115°09'E, 60 m a.s.l., 10.XI.–5.XII.2015, leg. A. Laciny & A. Kopchinskiy (“YG Vienna Colony”, specimen number COCY 01565).

**Paratypes** (59 minor workers, 8 major workers, 16 gynes, and 6 males dry mounted; > 500 imagines stored in 96 % ethanol): 19 minor workers, 2 major workers, 12 alate gynes, 4 dealate gynes, 6 males (including allotype) (all dry mounted), as well as 8 males, 2 alate gynes, ca. 500 minor workers (in alcohol) from the same nest sample as holotype; 1 major worker, same locality and collector as holotype, 17.IV.2015, “YG 373 main natural nest”; 1 major worker, same data as holotype (“YG doorkeeper #19”); 2 major workers, same data as holotype except 20.IV.2015, leg. A. Kopchinskiy (“cf. YG 39 (351) artificial nest”); 8 minor workers, 2 major workers, same locality as holotype, 2002, leg. D.W. Davidson (“YG KB02-108”); 4 minor workers, same locality and collector as previous, no collection date, “YG 2025”; 5 minor workers, same locality and collector as previous, I.2012, “YG T-trail (202)”; 5 minor workers, same locality and collector as previous, 15.V.2014, “YG-2 (73)”; 7 minor workers, same locality and collector as previous, 15.V.2014, “YG-2 (49)”; 2 minor workers, same data as previous except Batu Apoi Forest Reserve, N04°32', E115°10', 200 m a.s.l., 25.XI.2004, (“CAYG A-370”); 15 minor workers (on 5 pins), same data as previous, except N04°55', E115°19', 60 m, 3.VII.2002, (“YG KB02-108 voucher”); 4 minor workers, Thailand, Chumphon Province, Krom Luang Chumphon W.S, 3.II.2002, leg. W. Jaitrong (“WJT02-TH-0116”); 5 minor workers, West Malaysia, Kelantan, 60 km NE Tanah Rata, Tanah Kerajaan, 1000 m a.s.l., 12.–30.IV2007, leg. P. Cechovský.

Additional material: 3 pupae (Suppl. material 5) and 6 myrmecophilous crickets (*Camponophilus* sp., det. S. Ingrisch), from the same nest sample as the holotype.

For unique identification numbers of all 90 dry mounted specimens (60 minor workers, 8 major workers, 16 gynes, and 6 males), as well as information on caste and colony affiliation, see Suppl. material 6 “measurements”.

The holotype and a portion of the paratypes will be deposited at the Brunei Museum; additional paratypes will be housed in the Universiti Brunei Darussalam, the Natural History Museum Vienna, the University of California (Davis, USA), the Natural History Museum of Los Angeles County (Los Angeles, USA), the Thailand Natural History Museum (Technopolis, Thailand), and the collection of H. Zettel (Vienna, Austria).

## Molecular results

**Figure 1.**
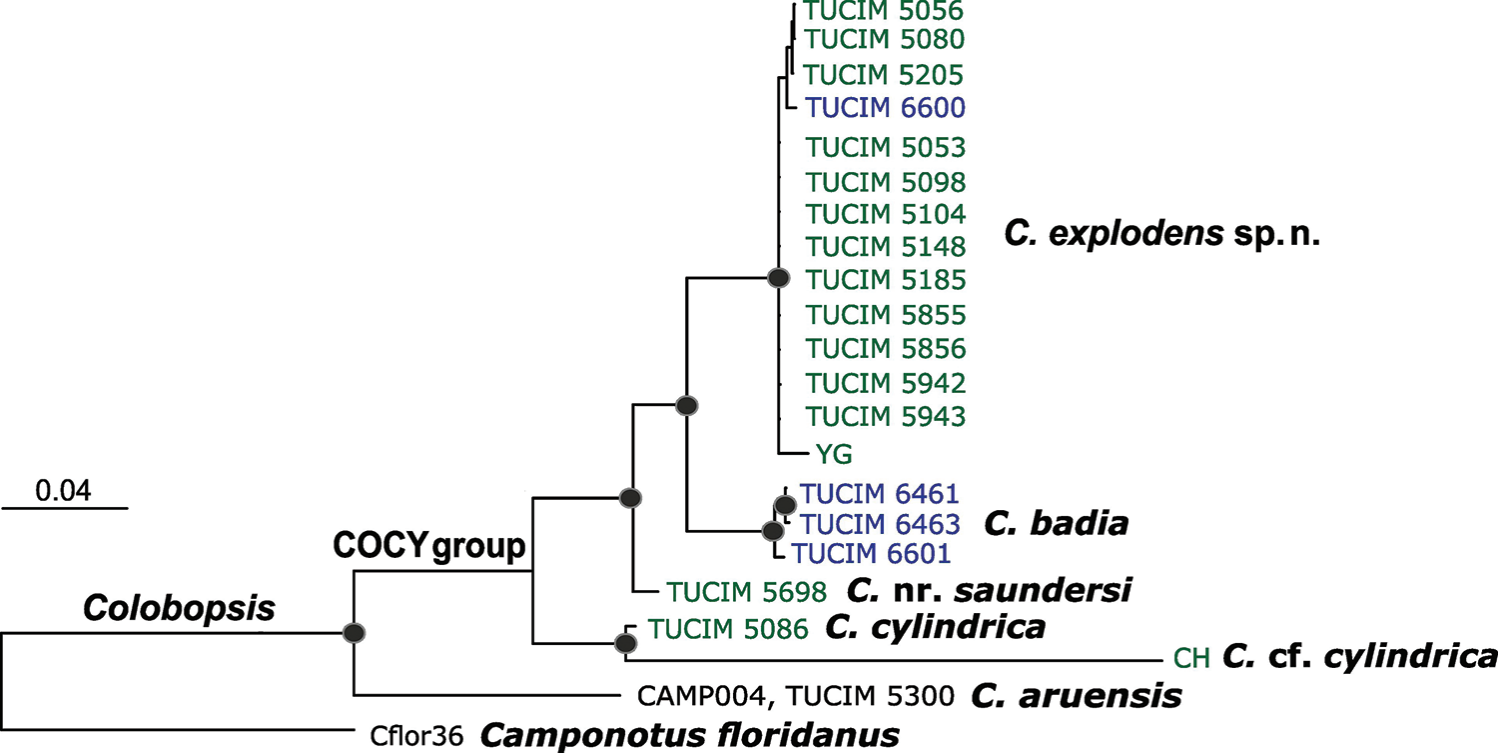
Bayesian consensus phylogram of *C. explodens* sp. n. and related taxa based on the concatenated alignments (2757 bp) of the three mitochondrial (COI, COII, cytB) and one nuclear (cad) phylogenetic markers. Nodes with posterior probabilities above 0.94 are marked with black dots. Specimens from Borneo are shown in green, from Thailand in blue. TUCIM 6463 corresponds to a male specimen.

The topology of the phylogram based on the concatenated alignment of 2757 bp was concordant with topologies of COI and COII and not contradicted by the topology of cytB. The phylogram based on cad was statistically unresolved (data not shown). The obtained Bayesian consensus tree (Fig. 1) shows conspecificity of the newly obtained *C. explodens* sp. n. specimens from Brunei and Thailand (TUCIM 6600) with a sequence previously deposited under “*Colobopsis cylindrica* s.l. YG”. While there is some intraspecific variation within the analysed *C. explodens* sp. n. specimens, they form a clade distinctly separate from the closely related *C. badia*. The male of *C. badia* (TUCIM 6463) is clearly grouped with its conspecific workers from a nearby locality, thus confirming species identity. The herein examined representatives of the *C. saundersi* subclade, *C. explodens* sp. n., *C. badia* and the undescribed *C.* nrSA (see Laciny et al. 2017), are clearly distinct from other members of the COCY group (e.g., *C. cylindrica*) and selected outgroup taxa of *Colobopsis* and *Camponotus*.

## Taxonomic results

### Colobopsis explodens

Taxon classificationAnimaliaHymenopteraFormicidae

Laciny & Zettel sp. n.

http://zoobank.org/DB4767B0-C745-4843-BE3F-B17DBCEB3A96

Figs 2 , 3 , 4 , 5 , 6 , 7 , 8 , 9 ; Suppl. materials 1 , 2 , 3 , 4 , 5 , 6 , 7

Camponotus (Colobopsis) sp. Yellow Goo: Davidson et al. 2007: 470.
Camponotus (Colobopsis) sp. YG: Cook 2008. Davidson et al. 2012: 488.
Colobopsis sp. YG: Davidson et al. 2016: 518. Laciny et al. 2017: 95.

#### Etymology.

Present participle of Latin *explodere*, referring to the “exploding”-like autothysis behaviour.

#### Description of phenotypes.

**Figure 2.**
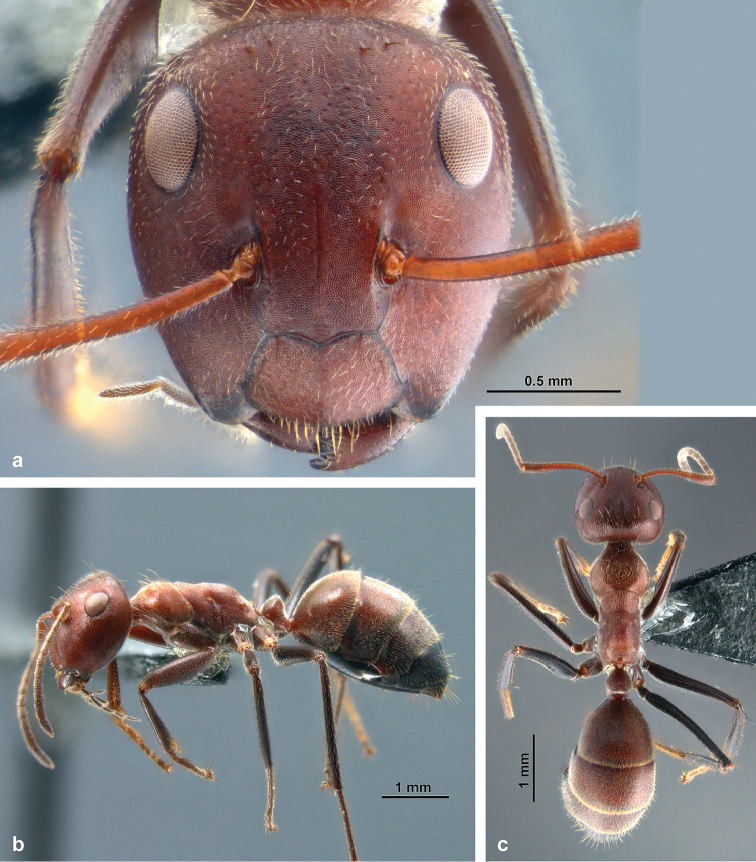
Habitus of *C. explodens* sp. n., holotype, minor worker; **a** full-face **b** lateral, and **c** dorsal view.

**Minor worker** (Figs 2, 4b–d; Suppl. material 1: S1a).

Measurements of holotype minor worker: TL 6.78; HW 1.48; HL 1.67; HS 1.58; PS5 0.23; PS6 0.25; EL 0.42; SL 1.33; SW 0.14; ML 2.05; HaL 0.15; PH 0.55; PL 0.47; NH 0.33; FeL 2.05. Indices: CI 88; SI 90; SWI 11; EI 29; PI 116; FeI 139; PSI 30.

Measurements of paratype minor workers: (n = 59): TL 4.74–7.21; HW 1.22–1.57; HL 1.30–1.78; HS 1.27–1.67; PS5 0.21–0.25 (20); PS6 0.20–0.26 (21); EL 0.33–0.43; SL 1.21–1.39; SW 0.11–0.16; ML 1.50–2.22; HaL 0.08–0.19; PH 0.41–0.56 (44); PL 0.33–0.49 (47); NH 0.24–0.38 (52); FeL 1.73–2.10. Indices: CI 85–94; SI 87–104; SWI 9–12; EI 27–29; PI 112–133 (41); FeI 123–151; PSI 28–35 (20).

*Structures*: Head (Fig. 2a) subovate, longer than wide, narrower anteriorly; sides posteriorly convex, posterior cephalic margin roundly convex; microstructure consisting of very fine, isodiametric or transverse mesh-like reticules; intermixed punctures very fine and inconspicuous on face, larger but shallow laterally and ventrally. Eyes small compared to other castes (EI 27–29, vs. 28–31 in major workers and 35–37 in gynes), flat, positioned dorsolaterally. Ocelli lacking, in some larger specimens position of median ocellus indicated by shallow impression. Frons with very fine impressed midline; frontal carinae slightly converging anteriorly, not elevated. Median carina of clypeus not reaching anterior clypeal margin, especially in small specimens. Mandibles mostly smooth, with rather dense punctures; masticatory margin with five teeth. Maxillary palpi long (PSI 28–35). Antennal scape long, its length roughly equal to head width (SI 87–104), moderately flattened, slightly widened towards apex, integument punctate. Antenna 12-segmented; antennal segment III approx. 1/5 shorter than each IV and V, and approx. 2/5 shorter than II. Mesosoma slender, moderately low. Microreticulation isodiametric or slightly transverse, dorsally denser than laterally. Metanotal region delimited from mesonotum by a shallow groove; groove delimiting metanotum from propodeum indistinct or missing. Dorsal and posterior outline of propodeum rounded in lateral view, or meeting at an obtuse angle, dorsal face slightly convex, posterior face flat to shallowly concave. Legs slender. Petiole with isodiametric reticulation; petiolar node moderately high, its short, slightly convex anterior and its rather straight posterior face forming a triangular shape in lateral view, its apex not truncated, rather rounded; node narrow in dorsal view, a crest indistinct; a medial depression indicated in most specimens. Gaster: dorsum of tergites I–III with extremely fine, dense, transverse microreticulation, slightly shiny (Fig. 4b); mesh-like reticulation wider on lateral areas of tergites I–III , tergite IV, and sternites , therefore meshes appearing not so strongly transverse, and the integument shinier (Fig. 4c). Exposed parts of tergite V and sternite V with dense, almost isodiametric reticulation, dull; base of tergite V (usually covered by tergite IV) sculptured as tergite IV.

**Figure 3.**
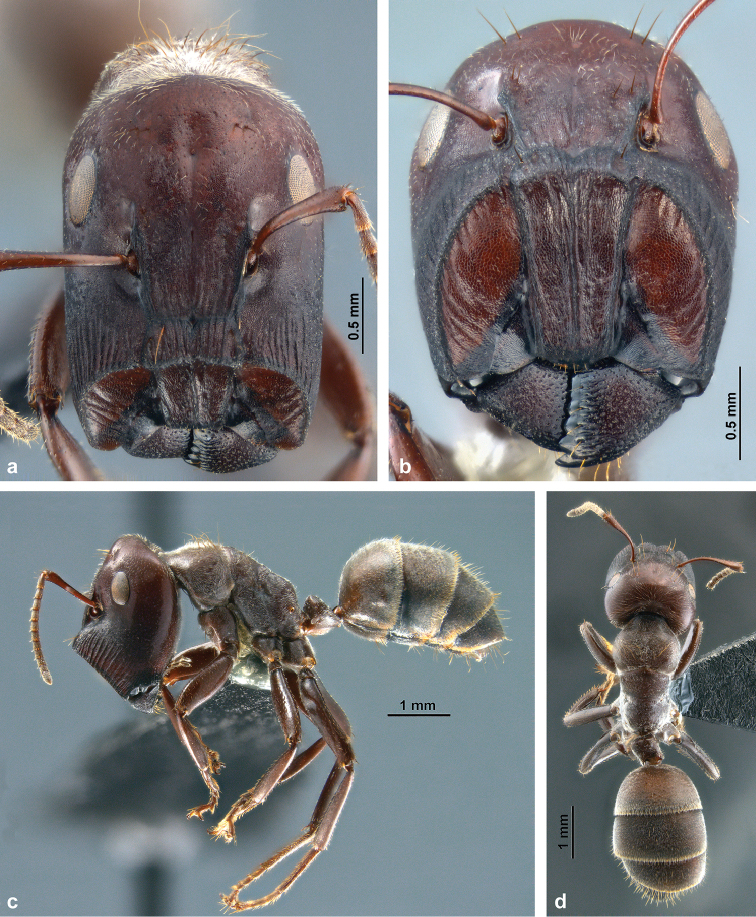
Habitus of *C. explodens* sp. n., paratype, major worker; **a** full-face view **b** frontal shield **c** lateral, and **d** dorsal view.

**Figure 4.**
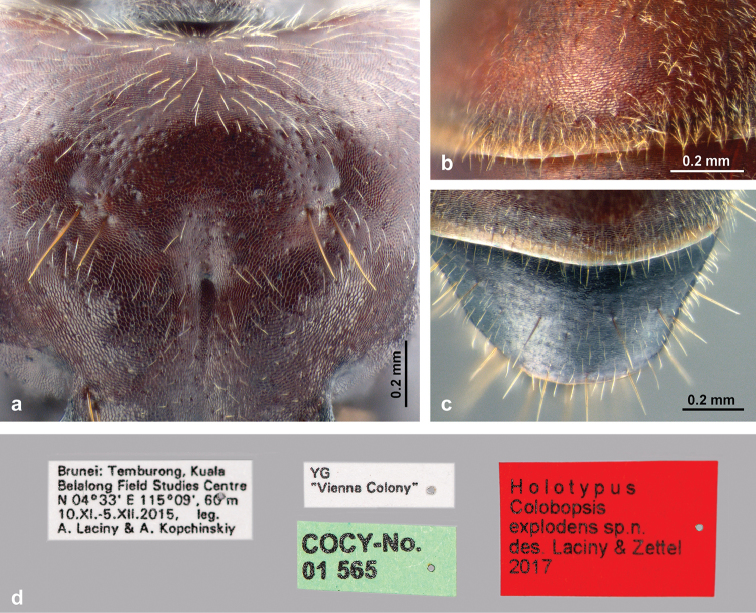
Cuticular microsculpture of *C. explodens* sp. n.; **a** vertex of major worker (paratype) **b** gastral tergite I, and **c** gastral tergite IV of minor worker (holotype) **d** labels of holotype, minor worker.

*Colour*: Body mainly reddish brown. Vertex of head, margins of clypeus, masticatory and lateral margins of mandibles, dorsum and ventral margins of mesosoma, mid portion of gastral tergites I–III, and legs slightly darker brown in most specimens; some specimens with darker area extending medially from head vertex to frons. Gastral tergites and sternites with very narrow hyaline margins. All gastral sternites, lateral fourths and posterior margins of tergites I–III, as well as entire tergites IV and V black.

*Pilosity*: Dorsum of head with very short, inconspicuous, appressed and subdecumbent setae; a few very long, standing setae on frons near declivity to vertex, medial of frontal carinae, and on lateral portions of clypeus. Mesosoma and petiole with fine and short, whitish, velvety pilosity; long, standing, slightly undulated setae restricted to pronotum; declivity of propodeum and node of petiole with few very short standing setae. Gastral tergites with moderately dense, short whitish, decumbent setae and few slightly darker, longer standing setae, most of them in transverse rows near hind margins. Longest setae in transverse rows near hind margins of sternites and at base of gastral tergite V.

*Notes*: Minor workers of *Colobopsis explodens* sp. n. show a continuous size variation across a remarkably wide range, similar to that found in the undescribed *Colobopsis* sp. nrSA (Fig. 8; compare with Laciny et al. 2017).

#### Phragmotic major worker

(Figs 3, 4a; Suppl. material 1: S1b).

Measurements of paratype major workers (n = 8): TL 7.30–8.71; HW 1.72–1.89; HL 2.25–2.58; HS 1.99–2.20; PS5 0.15–0.17 (6); PS6 0.15–0.17 (6); EL 0.50–0.56; SL 1.15–1.26; SW 0.17–0.20; ML 2.22–2.74; HaL 0.11–0.20; PH 0.59–0.69 (6); PL 0.45–0.51 (6); NH 0.40–0.45 (6); FeL 1.50–1.70. Indices: CI 71–77; SI 64–69; SWI 14–17; EI 28–31; PI 125–143 (6); FeI 87–95; PSI 14–17 (6).

*Structures*: Integument mostly dull, only head and legs shiny. Head (Fig. 3a) large, subcylindrical, anteriorly truncated. On posterior areas of face punctation slightly stronger than in minor worker. Eyes somewhat larger and more distant from vertex compared to minor worker. Ocelli lacking, their positions often indicated by shallow grooves (Fig. 4a). Anterior part of head forming a large shield (Fig. 3a, b) formed by clypeal and genal components, limited by a sharp and elevated crest so that the shield surface appears concave in lateral view. Shield with fine isodiametric reticulation and rather variable, mostly longitudinal rugae; most prominent are a pair of rugae along sides of clypeus and a single median carina that does not reach the anterior margin, often reduced towards base. Genal part with curved rugae of variable number, length, and distinctiveness, but only exceptionally reaching onto the anteromedial triangle. Additional longitudinal rugae on clypeus often present, including a usually distinct pair of paramedian rugae running from base of clypeus over the crest anteriorly towards middle of shield; in specimens with short median carina, the area between these carinae more or less grooved. Longitudinal striation more regular and pronounced on frons and genae up to level of antennal insertions, laterally on genae similarly long and strong. Mandible with sharp and high ventrolateral ridge, coarsely punctate, its lateral face weakly rugose-striate; masticatory margin with acute apex and few (1–3) more or less distinct, very blunt teeth in distal half (Fig. 3b). Maxillary palpi very short (PSI 14–16). Antenna considerably shorter than head width (SI 64–69) and stouter than in all other morphs (Figs 3a, 8b); antennal scape distinctly widened towards apex. Mesosoma stouter and higher than in minors, especially promesonotum expanded; in lateral view dorsal and posterior face of propodeum forming an obtuse angle, somewhat less rounded than in minor workers, dorsally without concavity. Legs much shorter and stouter than in minors (Fig. 3c). Shape of petiole similar to minor workers, somewhat wider in dorsal view. Structures of gaster similar as in minor worker.

*Colour*: Overall slightly darker than minor worker; head, legs and mesosoma reddish brown; gaster slightly darker chocolate-brown, becoming darker towards caudal apex, hyaline margins yellowish; elevated crest of frontal shield, anterior clypeal margin, frontal carinae, and masticatory and lateral margin of mandibles blackish brown.

*Pilosity*: As in minor worker, except long setae on clypeus sides restricted to the area behind clypeal shield; mesonotum with standing setae which are approx. half the length of those on pronotum.

*Notes*: The head shield with a sharp, elevated crest is typical for majors of the *Colobopsis saundersi* complex (Fig. 3b).

#### Gyne

**Figure 5.**
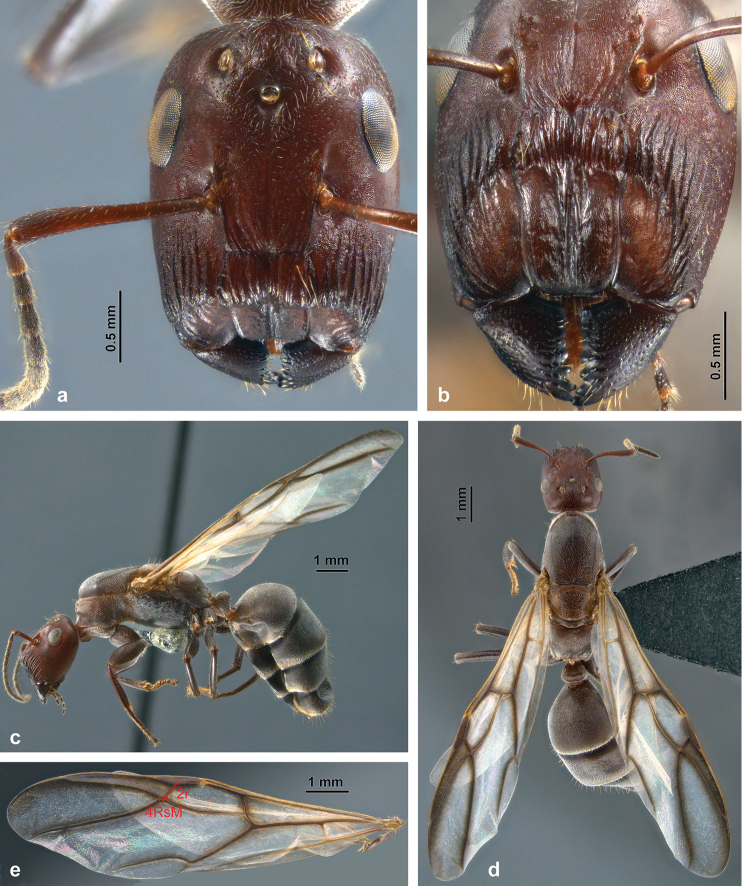
Habitus of *C. explodens* sp. n., paratype, alate gyne; **a** full-face view **b** frontal shield **c** lateral **d** dorsal view **e** forewing with indicated measurements 2r and 4RsM.

(Fig. 5, Suppl. material 3: S3d).

Measurements of paratype gynes (n = 16): TL 10.50–12.16; HW 1.74–1.83; HL 2.28–2.45; HS 2.02–2.14; PS5 0.19–0.21 (13); PS6 0.19–0.23 (13); EL 0.62–0.66; SL 1.33–1.45; SW 0.20–0.22; ML 4.11–4.63; HaL 0.14–0.29 (15); PH 0.77–0.92 (11); PL 0.54–0.67 (10); NH 0.40–0.54 (11); FeL 2.25–2.35; OcW 0.13–0.16; OED 0.34–0.38; OcD 0.54–0.64; FWL 9.72–10.50 (11); MSW 1.68–2.15; 2r 0.50–0.64 (12); 4Rs+M 0.14–0.32 (12). Indices: CI 73–77; SI 75–80; SWI 15–16; EI 35–37; PI 123–150 (8); FeI 125–132; PSI 18–20 (13); OI 54–69; WVI 26–58 (12).

*Structures*: Head (Fig. 5a) large, subcylindrical, anteriorly truncated, similar to that in major worker with the following exceptions: eyes larger than in workers (EI 35–37) and breaking outline of head in full-face view. Ocelli fully developed, their colour ranging from almost clear to reddish amber. Head shield sharply delimited, but slightly smaller than in major worker, distinctly narrower than head width. Striation of clypeus, frons, and genae similar as in major, though somewhat more strongly developed on lateral parts of shield. Mandible with sharp ventrolateral ridge; its lateral face weakly rugose-striate, narrower than in major; dorsal-anterior face punctured; masticatory margin with acute apex and 3–4 blunt teeth in distal half, mandible basally with blunt ridges (Fig. 5b). Maxillary palpi moderately long (PSI 18–20). Antennal scape moderately long, slightly shorter than head width (SI 75–80), somewhat widened towards apex (Figs 5a, 8b). Mesosoma large, structures as typical for caste; propodeum large and evenly convex in lateral view. Cuticular microstructures dorsally consisting of very fine punctation, with intermixed larger punctures, laterally finely reticulated. Legs stout, but not as short as in major (Fig. 5c). Forewing venation strongly reduced, as in most Camponotini; M-Cu absent; Mf2+ interstitial (Fig. 5e). Petiole distinctly wider than in workers; node more rounded in lateral view, in some specimens its apex shallowly impressed medially, in others with two shallow lateral impressions forming a trilobed outline. Gastral tergites I–IV and sternites I–IV with extremely fine and dense microstructures consisting of strongly transverse meshes; only sides of tergites with wide mesh-like reticulation and shiny; tergite V with dense isodiametric reticulation.

*Colour*: Chiefly as in major worker. Head and pronotum reddish brown; ventral and posterior mesosoma, petiole, legs and gaster somewhat darker chocolate-brown; mandibles and ridges of clypeal shield blackish brown. Pronotum and mesonotum with very narrow yellow margins. Gastral tergites medially with very narrow hyaline margins; sternites with relatively broad posterior margins. Wings hyaline, but forewing cells along veins, as well as pterostigma darkened to brownish. On hind wing all veins pale yellow (Fig. 5c–e).

*Pilosity*: Short pilosity and distribution of long setae on head, petiole, and gaster similar as in major worker, but that of mesosoma different; pronotum with few long, undulated setae. Medial part of mesonotum (between parapsidal furrows) with numerous long erect setae, scutellum with few long erect setae; lateral part of mesonotum in front of tegulae without setae.

*Notes*: The head shield with a sharp, elevated crest is typical for gynes of the *Colobopsis saundersi* complex (Fig. 5b).

#### Male

**Figure 6.**
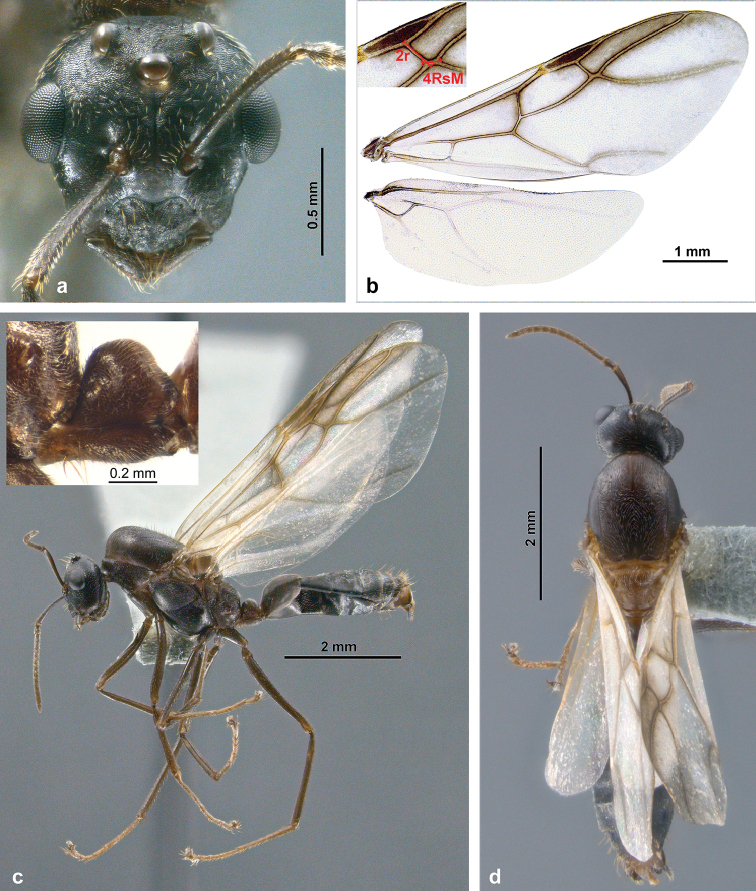
Habitus of *C. explodens* sp. n. allotype, male; **a** full-face view **b** wings (see insert for illustration of measurements 2r and 4RsM) **c** lateral (see insert for detailed view of petiole), and **d** dorsal view.

**Figure 7.**
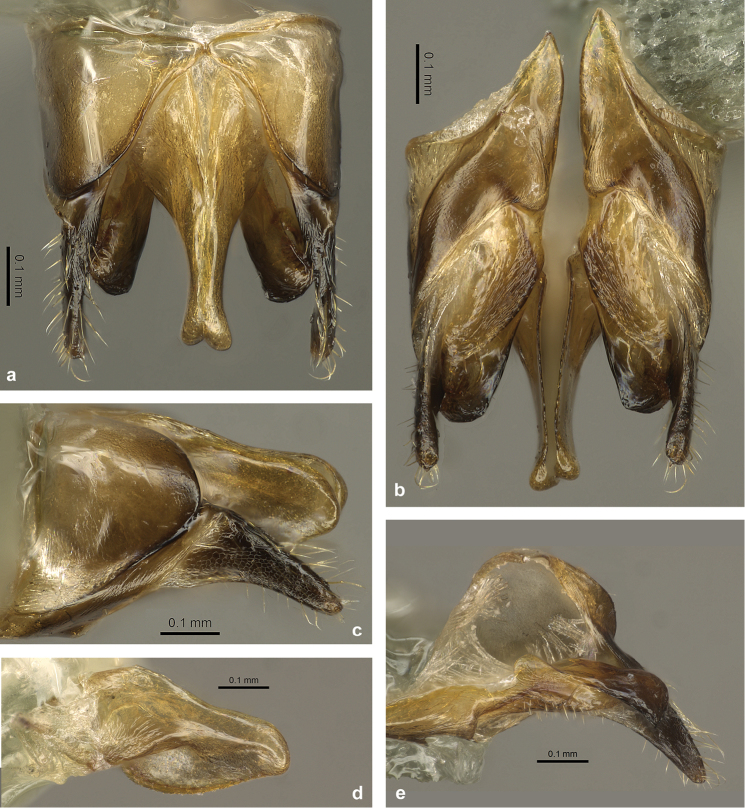
Genital structures of *C. explodens* sp. n. paratype, male; genital capsule in **a** dorsal **b** ventral, and **c** lateral view **d** left penis valve **e** right volsella and gonostylus.

(Figs 6, 7). This is the first detailed description and illustration of males from the *C. cylindrica* group.

Measurements of allotype male: TL 7.11; HW 1.26; HL 1.20; HS 1.23; PS5 0.20; PS6 0.15; EL 0.44; SL 0.84; SW 0.11; ML 2.54; HaL n.a.; PH 0.46; PL.40; NH 0.29; FeL 1.83; OcW 0.18; OED 0.27; OcD 0.43; FWL 6.33; MSW 1.37; 2r 0.38; 4Rs+M 0.27. Indices: CI 105; SI 66; SWI 13; EI 35; PI 116; FeI 145; PSI 28; OI 62; WVI 70.

Measurements of paratype males (n = 5): TL 6.46–6.85; HW 1.24–1.29 (4); HL 1.14–1.24; HS 1.20–1.27; PS5 0.17–0.21 (4); PS6 0.13–0.17 (4); EL 0.43–0.46; SL 0.80–0.85; SW 0.10–0.12; ML 2.38–2.87; HaL n.a.; PH 0.45–0.49 (4); PL 0.38–0.40 (4); NH 0.26–0.33 (4); FeL 1.71–1.86; OcW 0.18–0.19; OED 0.25–0.27; OcD 0.43–0.46; FWL 5.87–6.33; MSW 1.17–1.50; 2r 0.38–0.47; 4Rs+M 0.14–0.22. Indices: CI 104–110 (4); SI 64–67 (4); SWI 12–15; EI 35–36 (4); PI 113–123 (4); FeI 136–151 (4); PSI 27–30 (4); OI 53–62; WVI 31–53.

**Figure 8.**
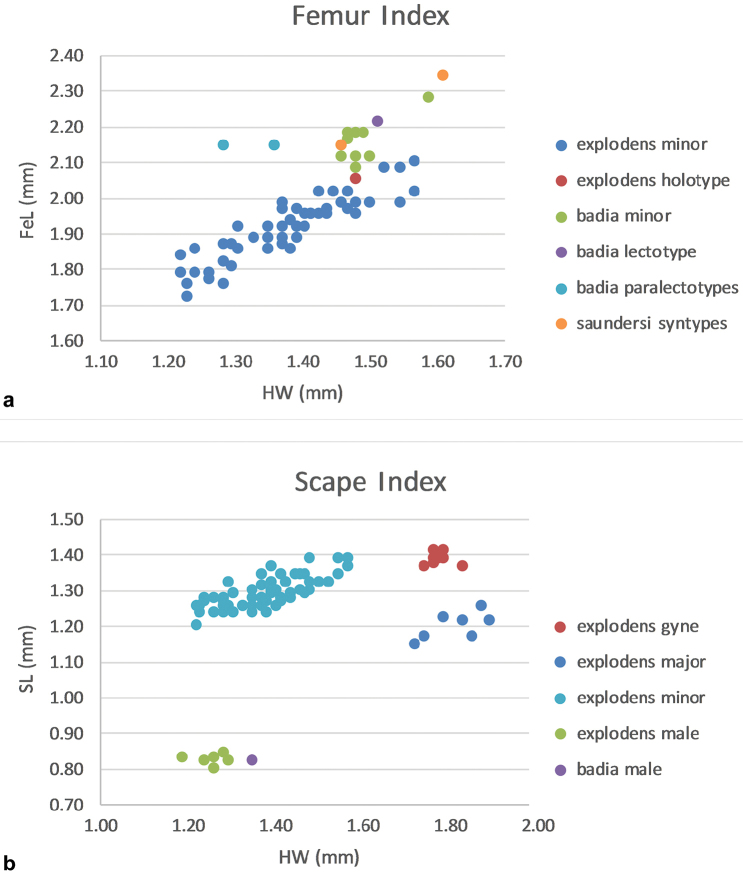
Variation of relevant morphometric measurements for the distinction of castes and species; **a** Metafemur length (FeL) in relation to head width (HW) for minor workers of *C. explodens* sp. n., as well as minor workers of *C. badia* and *C. saundersi* (type specimens plotted separately) **b** Scape length (SL) in relation to head width (HW) for minor workers, major workers, gynes and males of *C. explodens* sp. n., as well as male of *C. badia*.

*Structures*: Head (Fig. 6a) small, subtrapezoidal, eyes very large, round and protruding, EL more than one third of HL (EI 35–36). Ocelli very large, diameters larger than in gynes. Integument of head rather matt. Frons and genae finely reticulated, genae additionally finely punctured. Clypeus with some stronger punctures at margins (at base of setae), median carina weakly developed, present in proximal third of clypeus or entirely obsolete. Frons with impressed midline from median ocellus to level of antennal insertions. Frontal carinae weakly developed, converging more strongly than in minor worker. Mandible short with reduced dentition, masticatory margin with 2–3 blunt teeth; dorsal surface finely punctate. Maxillary palpi long (PSI 27–30). Antenna 13-segmented; scapes short (SI 64–67) and relatively slender (Fig. 8b). First funicular segment conspicuously enlarged distally, pear-shaped, 30–50% wider and ca. 20% longer than the following segment (Fig. 6a); all other funicular segments cylindrical, without modifications. Mesosoma large, structures as typical for alate ants. Mesoscutum anteriorly strongly convex with narrow impressed midline in posterior tenth. Scutellum moderately elevated; propodeum evenly convex. Cuticular microstructures of mesosoma consisting of a very fine reticulation with intermixed minute punctures at bases of short hairs, additionally with larger punctures dorsally at bases of erect setae. Legs very long and slender (FeI 136–151). Forewing venation strongly reduced, as in most Camponotini. M-Cu absent; 4Rs+M shortly developed or (more rarely) Mf2+ interstitial (Fig. 6b). Petiole small; in lateral view node more bluntly rounded than in female castes, anterior and posterior faces straight, not convex, apex not impressed medially in dorsal view. Gastral tergites I–IV and sternites I–IV with fine and dense microreticulation consisting of moderately transverse meshes; only sides of tergites with wide meshes and shiny; tergite V with almost isodiametric reticulation. Sternite VI posteriorly emarginated, sternite VII truncated.

*Genital structures* (Fig. 7): Genital capsule (Fig. 7a–c) approx. as long as wide in dorsal aspect (Fig. 7a), ventrally longer than dorsally, protruding from apex of gaster. Gonopod high, distally broadly rounded. Gonostylus (Fig. 7c) elongated and acuminated, with reticulated microstructure (only visible at very high magnification) and some long setae. Basivolsella (Fig. 7e) dorsally with roundish structure, ventrally with evenly distributed, comparatively short setae. Digitus (Fig. 7e) large, evenly widened towards apex; apex rounded but with rectangular corner ventrally. Penis valvae (Fig. 7d) in dorsal aspect broad at base, but very narrow distally. Valviceps leaf-shaped in lateral view, apically rounded; surface smooth; ventral margin with very fine serration.

*Colour*: Mainly dark chocolate-brown. Head somewhat darker; eyes pale grey to blackish; ocelli translucent, ranging from almost clear to reddish amber. Antennae and legs lighter brown, fading into yellowish towards apices. Margins of mesoscutum, scutellum, and metanotum lighter yellowish brown. Gastral tergites medially with very narrow hyaline margins; sternites with relatively broad, indistinctly separated posterior margins. Wings almost hyaline, with a slight whitish tinge, but forewing cells along veins, as well as pterostigma darkened to brownish, all veins pale yellowish brown. On hind wing all veins pale yellow.

*Pilosity*: On head setae sparsely distributed, short, inconspicuous, appressed, subdecumbent; a few very long standing setae on frons near vertex, and on anterior and posterior clypeal margins. Mandibles with dense short pilosity on lateral face, and few moderately long setae on anterolateral margin. Short pilosity and distribution of long setae on mesosoma, petiole, and gaster similar as in gyne, but pronotum lacking long, undulated setae. Medial part of mesonotum (between parapsidal furrows) with numerous long erect setae, scutellum with few long erect setae; lateral part of mesonotum in front of tegulae without setae. Tegulae with dense brush of setae. Petiole with a few stout setae anteroventrally (see insert Fig. 6c). Petiolar node lacking any standing setae; gastral tergite I without or with few subdecumbent, moderately long setae. Posterior gastral tergites and sternites (segments II and following) with sparse, relatively long, obliquely standing setae.

##### Biological notes on *Colobopsis explodens* sp. n.

Colonies of *C. explodens* sp. n. observed in the Ulu Temburong National Park are commonly polydomous and polygynous. This species was selected as a model for the study of the “exploding ants” because among the species with advanced autothysis behaviour it was the most abundant COCY taxon in the vicinity of KBFSC.

*Colobopsis explodens* sp. n. frequently nests on dipterocarp trees and its colonies can contain thousands of individuals. The largest part of the studied colony lived on a 60 m tall *Shorea johorensis* Sym. (Dipterocarpaceae) tree identified morphologically and by DNA barcoding (matK, identical to GenBank accession number KY973022, E-value is zero; Heckenhauer et al. 2017). The colony’s foraging ground included the canopy of the main tree, its direct vicinity, and also covered canopies of a 25 m tall *Horsfieldia wallichii* (Hook.f. & Th.) Warb. (Myristicaceae) tree and a smaller tree of *Shorea maxwelliana* King (9 m). Colony fragments on all trees were connected by ant trails either through the canopy or on the forest floor in the litter layer. The total area occupied by the colony was estimated to be at least 2500 m^2^.

The colonies are distributed three-dimensionally, occupying any suitable nesting ground within the colony boundaries. On the main tree, we found four nesting sites of the examined colony in dead branches at heights ranging between 35 and 55 m above ground and two nesting sites in the living stem 50–60 m above ground. No nests in living branches were observed. At least five nest entrances were also seen in the stem of *S. maxwelliana*. No signs of necrosis of the plant tissue were observed around stem entrances.

The translocation of a nest fragment in a fallen branch to the laboratory’s terrace, 30 m away from its original location, resulted in the expansion of the colony’s foraging ground to a neighbouring *Shorea* sp. tree where these ants were not previously present, while the connection to the colony on the original host tree was maintained.

**Figure 9.**
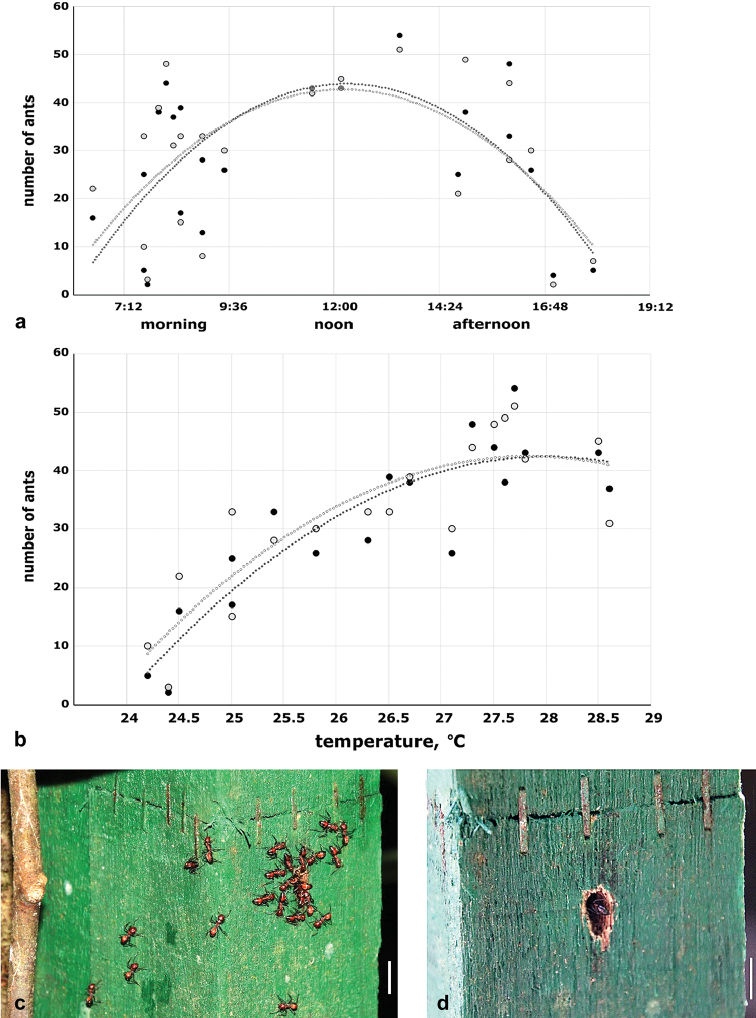
Activity of *C. explodens* sp. n. at the entrance to the artificial nest #38. **a** Scatter plot of the number of minor workers entering (black dots) and leaving (open dots) the nest depending on the time of day **b** Scatter plot of the number of minor worker ants entering (black dots) and leaving (open dots) the nest depending on the air temperature. Polynomial trend lines on a and b are shown for the numbers of entering (black) and exiting (grey) minor workers **c** and **d** show high and low worker activity near the entrance of nest #38, respectively; white bars denote 1 cm.

If provided with an appropriate artificial nest (Fig. 9c–d), *C. explodens* sp. n. ants will inhabit it within several weeks up to several months and even use it to rear brood. One artificial nest, mounted on the main host tree, was colonized one week after it was installed. For the activity assessment, the easily accessible artificial nest #38 was observed. During behavioural monitoring, *C. explodens* sp. n. was observed to be mainly diurnal, foraging between 6:00 and 18:00 hrs, with peak activity around 9:00 and 16:00 hrs (Fig. 9a). The activity correlated positively with the temperature with lowest values at 24.2 °C and highest at 28.6 °C (Fig. 9b). The atmospheric pressure and clouds did not influence the activity of *C. explodens* sp. n. (Suppl. material 6 “activity”); humidity was constant over the period of observations ranging from 86 to 88%. A slight rain on a warm day did not reduce the activity of ants near the nest but no activity was observed during heavy rains. However, if a shelter was provided, *C. explodens* sp. n. remained active also during the rain and even after sunset. A drastic reduction in the number of minor workers at the nest entrance was observed on the days of nuptial flight, when several alate gynes and males left the nest in the early evening (Suppl. material 6 “activity”). Between one and six minor workers (“guards”) were frequently positioned at the nest entrance, touching all incoming and outgoing workers with their antennae and seemingly monitoring the activity of foragers. In the early afternoon of the day with the highest activity, larvae were carried out of the nest. No carrying of larvae into the nest was ever observed.

Remarkably, during all observations the numbers of the minor workers leaving and entering the nest were almost equal. The fact that this proportion did not change over the day (Fig. 9a) suggests a tendency to maintain a constant number of individuals present inside the nest.

After dusk, other species of Camponotini such as *Polyrhachis* spp., *Camponotus* spp., and *Dinomyrmex gigas* (Latreille, 1802) were observed on the trees in the vicinity of the artificial nest.

Within the colonies, minor workers were by far the most abundant caste of *C. explodens* sp. n., whereas major workers (soldiers) were rare and almost never seen outside the nest. Alate gynes and males were observed leaving the nest during nuptial flight after dusk on two occasions during our field observations (Suppl. material 6 “activity”). Several more alate sexuals were found inside a detached nest fragment (Suppl. material 3). The same nest fragment also contained symbiotic ant crickets of the genus *Camponophilus* Ingrisch, 1995. DNA barcoding of the cricket based on COI sequence resulted in the highest value of 80 % similarity to COI sequences of insects from several groups including Mann’s ant cricket *Myrmecophilus manni* (Schimmer, 1911) (EU938370, Fenn et al. 2008). Thus, the molecular identification of these crickets is currently not possible due to lack of reference sequences.

Observations have shown that minor workers of *C. explodens* sp. n. display a characteristic, possibly defensive pose with raised gaster (Suppl. material 1: S1a) (compare with Davidson et al. 2007) and are extremely prone to self-sacrifice when threatened. The mandibular gland content is released during autothysis by contracting the gaster until the integument ruptures, leading to the death of the ant (Suppl. material 4). The secretion is slightly viscous, sticky, and has a species-specific bright yellow colour and a distinctive spice-like odour (Hoenigsberger et al., in prep.).

Minor workers of *C. explodens* sp. n. spend significant time on leaves, which has previously been hypothesized to contribute to their nutrition (compare with Davidson et al. 2007, 2016). However, the exact purpose of their activity on leaves is yet to be understood. Observations suggest a patrolling or monitoring behaviour aiming at the removal of debris from the phyllosphere (mainly adaxial leaf surface but also abaxial leaf and petiole surfaces) and possible deterrence of intruding arthropods. Similar “cleaning” behaviour was observed *in vitro*, as well.

Another very specific behaviour was exhibited on the tree bark: Minor workers “graze” on the layers of epiphytes (mosses, lichens, algae, filamentous fungi, and yeasts) with their mandibles, often for up to 60 minutes. This behaviour differs from the cleaning behaviour on leaves and presumably contributes to the ants’ nutrition.

Preliminary feeding experiments using cultures of filamentous fungi isolated from the phyllosphere of the host trees remained unsuccessful, no fungal feeding was observed. Only a suspension of yeast in water was accepted *in vitro* (M. Rahimi, pers. obs.). However, minor workers of *C. explodens* sp. n. have been observed to feed on small dead insects, fruit, and fish when offered on the foraging grounds (A. Kopchinskiy, A. Laciny & M. Hoenigsberger pers. obs.).

Commonly observed modes of behaviour of *C. explodens* sp. n. *in situ* and *in vitro* as well as a variety of nesting sites are documented in the Suppl. material 7 (Video S7).

The molecular analysis of the mandibular gland (MG) content of *C. explodens* sp. n. resulted in PCR amplification and sequencing of the 16S rRNA fragment of the bacteria *Blochmannia* sp. (Gammaproteobacteria), a genus of obligate symbiotic bacteria found in carpenter ants (Williams and Wernegreen 2015). We revealed four identical mOTUs originating from two different DNA extracts from samples composed of five pooled MG reservoirs of the minor workers each. The sequences of 728 nt were 99 % similar (11 SNP sites) to the “uncultured bacterium clone 193-11” KC136854 from *Camponotus* sp. voucher KC-A017-01 defined as *Blochmannia* sp. in Russell et al. (2012).

More detailed data on autothysis, composition of mandibular gland secretion, biodiversity of the COCY-associated microorganisms, and experimental assessment of nutrition will be presented in future publications.

### Colobopsis badia

Taxon classificationAnimaliaHymenopteraFormicidae

(Smith, 1857)

Figs 8 , 10

Formica badia : Smith 1857: 54.
Camponotus badius : Roger 1863: 3.
Colobopsis badia : Ward et al. 2016: 350. Bolton 2017.

#### Type material

examined. 1 lectotype minor worker (Oxford University Museum of Natural History, present designation), Singapore, “Formica badia”, “Syntype”, CASENT 0901897, “Lectotypus Formica badia Smith, 1857 des. Laciny & Zettel, 2017”, 2 paralectotype minor workers (Oxford University Museum of Natural History) mounted on the same card, Sarawak (“Sar 32”), “Formica badia”, “Syntype”, “Paralectotypes Formica badia Smith, 1857”.

#### Additional material examined.

1 male (Natural History Museum Vienna), Thailand, Trang Province, Nayong District, Khao Chong Botanical Garden, at light of head quarter, 7°33'N, 99°46'E, 60 m a.s.l., 1–7.VI.2016, leg. H. Zettel (68); 10 minor workers (Natural History Museum Vienna), Thailand, Trang Province, Nayong District, Khao Chong Botanical Garden, trail to Ton Pliw Waterfall, N07°32'34", E99°47'33", 150 m a.s.l., 1–7.VI.2016, leg. H. Zettel (66-4).

#### Description notes on the type specimens.

*Lectotype*: Minor worker glued to a square cardboard, in relatively good condition; right hind leg missing; tarsi of middle legs and left hind leg broken; erect setae on dorsum probably lost. Structures agree well with other species of the *C. saundersi* complex, a few characteristic features are given: Setae on scape more decumbent than in *C. explodens* sp. n. Dorsal outline of mesosoma almost straight, only with weak indentation at meso-metanotal suture. Propodeum forming a distinct obtuse angle in lateral view. Petiolar node relatively short, apex acute in lateral view, its crest slightly indented in middle. Tergites I–III with very fine, strongly transverse microsculpture (lateral parts not visible). Colour relatively dark brown; appendages strongly infuscate; antennal segments III–XII, meso- and metafemora almost black.

*Paralectotypes*: Two minor workers glued to the same square cardboard, in relatively poor condition. Left specimen with damaged head and gaster, lacking right middle leg; major parts of body covered by dirt or glue; most erect setae probably lost. Right specimen with slightly damaged head, lacking gaster and right hind leg; some parts of body covered by dirt or glue; most erect setae probably lost. The two specimens are probably conspecific, but conspecificity with the lectotype is uncertain. The combination of morphological features is intermediate between *C. badia* and *C. explodens* sp. n.: setae on scape similar to *C. explodens* sp. n., more erect than in the lectotype; dorsal outline of mesosoma intermediate, more structured than in the lectotype, but propodeum with angle; shape of node intermediate, apex more acute than in *C. explodens* sp. n. Colour almost as dark as in the lectotype.

Measurements of lectotype minor worker: TL 6.13; HW 1.51; HL 1.63; HS 1.57; PS5 n.a.; PS6 n.a.; EL 0.40; SL 1.43; SW 0.15; ML 1.96; HaL 0.17; PH 0.54; PL 0.36; NH 0.32; FeL 2.22. Indices: CI 93; SI 95; SWI 10; EI 26; PI 150; FeI 147; PSI n.a.

Measurements of paralectotype minor workers* (n = 2): TL 6.13, n.a.; HW n.a., 1.36; HL n.a., 1.52; HS n.a., 1.44; PS5 n.a., 0.21; PS6 n.a., 0.25; EL 0.36, 0.37; SL 1.39, 1.40; SW 0.12, 0.13; ML 1.89; HaL 0.13, n.a.; PH n.a., 0.51; PL 0.37, 0.42; NH 0.27; FeL 2.15. Indices: CI n.a., 89; SI n.a., 103; SWI 8, 9; EI n.a., 27; PI n.a., 121; FeI n.a., 158; PSI n.a., 32. *One specimen with strongly damaged head, one with missing gaster.

Measurements of non-type minor workers (n = 10): TL 5.64–6.23; HW 1.46–1.59; HL 1.63–1.72; HS 1.54–1.65; PS5 0.24–0.25 (3); PS6 0.24 (3); EL 0.38–0.40; SL 1.37–1.43; SW 0.13–0.14; ML 1.96–2.22; HaL 0.13–0.19; PH 0.51–0.56 (7); PL 0.41–0.45; NH 0.31–0.37 (9); FeL 2.09–2.28. Indices: CI 88–92; SI 90–96; SWI 9–10; EI 25–27; PI 118–130 (7); FeI 141–148; PSI 30–31 (3).

#### Male.

*Notes on collecting and identification*: A single male collected at light was identified as a specimen of the *C. cylindrica* group. DNA barcoding revealed specific identity with a nest series of *C. badia* from the same botanical garden. The morphological identification of this nest series (Col. 66-4) was carried out by direct comparison to the lectotype of *C. badia*.

**Figure 10.**
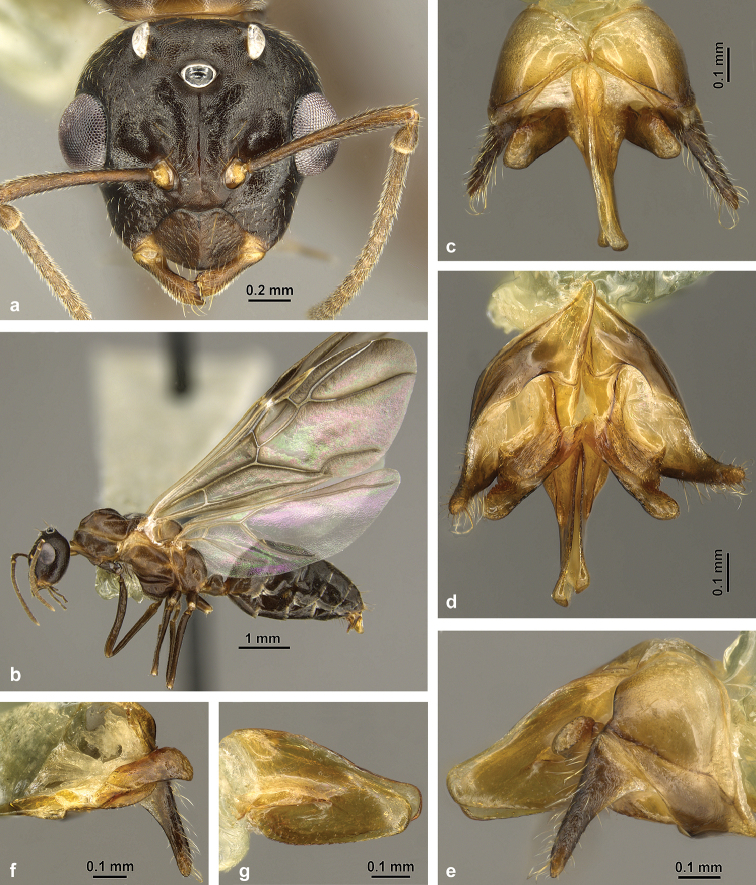
Habitus of *C. badia*, male; **a** frontal **b** lateral; genital capsule in **c** dorsal **d** ventral, and **e** lateral view **f** right volsella and gonostylus **g** left penis valve.

*Description* (Fig. 10): Overall very similar to *C. explodens* sp. n. and differing by the following characters:

Measurements of male (n = 1): TL 8.28; HW 1.35; HL 1.26; HS 1.30; PS5 0.20; PS6 0.15; EL 0.48; SL 0.83; SW 0.10; ML 3.07; HaL n.a.; PH 0.47; PL 0.41; NH 0.31; FeL 1.96; OcW 0.19; OED 0.29; OcD 0.46; FWL 7.43; MSW 1.37; 2r 0.41; 4Rs+M 0.37. Indices: CI 107; SI 61; SWI 13; EI 36; PI 116; FeI 145; PSI 26; OI 61; WVI 91.

*Structures*: Size larger (TL ca. 8.3 mm). Integument rather shiny (Fig. 10a, b), especially on mesosoma. Clypeus with distinctly developed median carina, almost reaching anterior margin. Maxillary palpi (PSI 26) and antennal scapes (SI 61) relatively short. First funicular segment slightly more enlarged (30% wider than the following segment, Fig. 10a). Vein 4Rs+M of forewing long. Petiolar node slightly more widely rounded in lateral aspect.

*Genital structures* (Fig. 10c–g) very similar to *C. explodens* sp. n., with the following exceptions: Gonostylus very narrow, with weaker reticulation of lateral surface (Fig. 10e). Basivolsella with extremely short ventral setae (Fig. 10f). Digitus with rounded apex, without ventroapical corner (Fig. 10f). Valviceps with slightly coarser ventral serration (Fig. 10g).

*Colour*: Head chiefly dark brown, with lighter area comprising frons between antennal insertions and clypeus. Eyes grey. Ocelli clear, almost colourless. Posterior and anterior clypeal margins, as well as proximal fourth of clypeal carina black. Gaster dark brown. Mesosoma, petiole, mandibles, antennae, and legs lighter brown, appendages becoming yellowish towards apices. Antennal insertions, mandibular bases, margins of thoracic sclerites (especially below tegulae) creamy yellow. Gastral tergites medially with very narrow hyaline margins; sternites with relatively broad posterior margins. Wings hyaline, forewing with a slight brownish tinge and cells along veins, as well as pterostigma darker brownish, all veins pale brown. On hind wing all veins pale yellow.

*Pilosity*: Appressed and subdecumbent setae comparatively shorter and sparser, but difference less obvious on gaster. Standing setae on mesonotum and gaster shorter, on mesonotum less numerous.

*Comparative notes*: The male of *C. badia* can be distinguished from males of *C. explodens* sp. n. by larger body size, differing colour pattern, more shiny integument, well-developed clypeal carina, differing proportions of wing venation, and relatively shorter scapes (Fig. 8b). In the genitalia, the most striking differences are in the narrower gonostylus and the more rounded digitus apex (compare Figs 7e, 10f).

## Discussion

### Molecular results

In this study, three mitochondrial DNA loci and one nuclear DNA fragment were applied for the construction of a molecular phylogenetic tree (Fig. 1). The evolutionary analysis based on four loci showed that *C. explodens* sp. n. is clearly genetically distinct from morphologically similar species. A minor level of infraspecific polymorphism within the specimens from Brunei was observed for COI, COII, and in particular for cytB marker. While tree topologies based on single mitochondrial loci were concordant, the cad tree (nuclear locus) was not resolved for the entire COCY group. The comparison of COI sequences with several hundred COCY sequences available in our local database and 13 sequences deposited in NCBI (Nov. 2017) suggests that this marker can be used for the reliable molecular identification (DNA barcoding) of *C. explodens* sp. n., as COI sequences of the nearest COCY taxa share only 91% similarity (*C. badia*), and the similarity to the selected non-COCY species *C. aruensis* is 83%.

The DNA extraction from the gastral parts of the mandibular gland reservoirs of *C. explodens* sp. n. minor workers resulted in drastically low yields indicating no abundant microbial symbionts present there. The successful 16S rRNA PCR amplification gave a sharp band that was sequenced with high reproducibility. The 16S rRNA fragment corresponding to the whole genome sequenced *Blochmannia* endosymbiont of North American *Colobopsis obliquus* strain 757 (NCBI GenBank accession number CP010049, Williams and Wernegreen 2015) was 92 % similar to mOTU revealed in this study resulting in 56 polymorphic sites. This confirms that *C. explodens* sp. n. also harbours these bacteria that usually colonize the midgut of Camponotini workers (Sauer et al. 2002) and are considered to be beneficial for N-nutrition of these ants; they may also contribute to the general health of the workers and gynes. Thus, the detection of cf. *Blochmannia* bacteria rather indicated the contamination of the MG sample by fragments of the digestive system. In this respect, it is interesting to note that no *Wolbachia* (Alphaproteobacteria) mOTUs were recovered, but neither digestive tract nor ovaries were specifically investigated.

### Taxonomy

The treatment of *Colobopsis* as a genus separate from *Camponotus* is supported by molecular, morphological, and biological data (Blaimer et al. 2015, Ward et al. 2016). Naked pupae (Wheeler 1904; see Suppl. material 5) and presence of phragmotic soldiers and gynes are important features of *Colobopsis*, although unknown in many of the 94 valid species assigned to this genus by Ward et al. (2016). The morphological separation of minor workers of *Colobopsis* and *Camponotus* is chiefly based on head morphology, but complicated by extensive evolutionary changes within each group (Ward et al. 2016); however, the phylogeny of *Colobopsis* species has not been studied to date. The molecular data published by Blaimer et al. (2015), obtained from only four species, do not allow an interpretation of the relationships of intrageneric clades. Attempts to classify the species by morphological characters (Emery 1925, McArthur 2012), although useful for a rough sorting of species, probably hardly reflect their evolutionary relationships.

A first attempt of a comprehensive classification of the species of *Colobopsis* (as a subgenus of *Camponotus*) was done by Carlo Emery. In his outstanding treatment of Formicinae (Emery 1925) he treated 58 species and established six groups to hold 49 of them (nine remained unclassified). He defined the [Camponotus (Colobopsis)] *cylindricus* group by a gradual variation between worker and soldier, interspecific variation of head in soldiers and females (from concave and marginate to oblique and obtuse), and generally large size. Emery included eight species presently classified as *Colobopsis* (Ward et al. 2016), of which *Colobopsis calva* Emery, 1920, *C. quadriceps* (Smith, 1859), and *C. smithiana* (Wheeler, 1919) are not presently assigned to this group (see below), whereas *C. badia* and *C. corallina* were not included (listed under incertae sedis). Although Emery (1925) correctly recognized the size variation of workers, he failed to recognize the unique characteristics of the soldier caste (see Laciny et al. 2017).

More species of the COCY group were subsequently described by Stitz (1925), Menozzi (1926) and Karawajew (1929, 1935). A second attempt at classification was made by McArthur (2012): His Camponotus (Colobopsis) cylindricus group consists of species with “neck attached to head well below vertex” and is broader than Emery’s (1925) *cylindricus* group. It includes the following species (according to current classification) that do not fit the characteristics of the COCY group in the present sense: *Colobopsis anderseni* (McArthur & Shattuck, 2001), *C. brachycephala* Santschi, 1920, *C. cotesii* (Forel, 1893), *C. desecta* (Smith, 1860), *C. excavata* (Donisthorpe, 1948), *C. hosei* (Forel, 1911a), *C. mutilata* (Smith, 1859), and *C. quadriceps*, as well as *Camponotus dedalus* Forel, 1911b, and *Camponotus kutteri* Forel, 1915.

According to our morphological studies the COCY group can be defined as such: polymorphic *Colobopsis* with at least three distinct female castes: (i) winged, phragmotic gynes, (ii) phragmotic soldiers (doorkeepers), and (iii) minor workers with a considerable size variation; intermorphic workers may occur in addition (Laciny et al. 2017). Minor workers: Vertex highly raised above foramen. Eyes of minor worker small and flat, not breaking head sides in full-face view. Entire trunk with dense, reticulated microstructures; punctures of integument often reduced. Head with moderate, mesosoma with dense pubescence. Mesosoma (at least the pronotum, except in *C. clerodendri* Emery, 1887) dorsally with long undulated setae, never arranged in distinct rows. Gaster with appressed pubescence and two or three types of setae of different lengths (not arranged in rows, except at hind margin). Soldiers (not known of all taxa): differing from minor workers by enlarged heads and short appendages (antennae, palpi, legs); in most species with a clearly circumscribed head shield for phragmosis. Microsculpture and pilosity similar to minor worker.

Following this definition, the COCY group presently comprises 17 names in the rank of species, subspecies or variations, which are partly in synonymy to each other. The strong intraspecific variation of minor workers, the frequently lacking knowledge on soldiers (or gynes), and the historical descriptions of taxa from different morphs (either workers or gynes) make the species taxonomy a true challenge. A preliminary analysis of morphological and molecular data (unpublished) supports the division of the group into four species complexes (molecular data of one complex not available). We restrict the following analysis to the species of the *C. saundersi* complex, which includes *C. explodens* sp. n. and can be defined by the following combination of characters observable in minor workers and soldiers: head always red or brown (not black); mesosoma moderately elongated and dorsally with some long undulated setae, at least on pronotum; node of petiole without long setae; gastral tergites with dense (in most species strongly transverse) micro-reticulation and with small hair pits (without large grooves). Soldiers and gynes (not known of all species) have a strongly truncated head, in most species with a well-defined (crested) head shield. The following names are available in this group (listed chronologically): *Colobopsis badia* (Smith, 1857), *C. corallina* Roger, 1863, *C. saundersi* Emery, 1889, C. badia var. krama Forel, 1912, *C. badia saginata* Stitz, 1925, *C. solenobia* (Menozzi, 1926), and *Colobopsis trieterica* (Menozzi, 1926), comb. n.

*Colobopsis corallina* (=*C. solenobia* syn. n., =*C. trieterica* syn. n.) is an endemic species from the Philippines. Soldiers and gynes differ strongly from *C. explodens* sp. n. and other taxa of the complex (as far as such morphs are known) by a very obtusely margined, not crested head shield. Minors have a bright orange colour on head, mesosoma, and petiole, often extending to gastral tergite I. Morphometrically, the examined minor workers of *C. corallina* (n = 31) mainly differ from those of *C. explodens* sp. n. by a greater average length of appendages (SI 92–109 vs. 87–104; FeI 136–159 vs. 123–151; PSI 30–39 vs. 28–35).

The greatest similarity is observed between *C. explodens* sp. n. and *C. saginata* (stat. n.), a taxon only known from a single alate gyne from Northern Borneo. The important structures of the head shield are almost identical. Although strongly different from *C. explodens* sp. n. by pale orange brown colour, this gyne differs only by subtle morphometric characters of which the long and distally wide scape seems to be the most reliable (SI 83 vs. 78–80). The length of vein 4Rs+M is considerably longer in *C. saginata* than in *C. explodens* sp. n. (WVI 65 vs. 26–58).

Colobopsis badia var. krama, described from a soldier from Java (Forel 1912) is a very poorly known taxon. We have not been able to study any further material from Java yet. The type (illustrated by AntWeb.org under CASENT0910610) differs from *C. explodens* sp. n. by a pale red head that strongly contrasts with the dark brown mesosoma, by a well-developed median carina of the head shield that reaches the foremargin of the clypeus, and by a stronger punctation of the preocellar area.

*Colobopsis badia* was described by Smith (1857) from Singapore and Sarawak (Borneo). However, the original description is too brief to draw any meaningful taxonomic conclusions. Viehmeyer (1916) describes workers of this species in more detail, also noting the secretion of a sticky liquid upon capture. He mentions a strong variability in colouration (from red to almost black with reddish head) and propodeal shape. This raises the question whether all examined specimens were truly members of the same species or perhaps belonged to one of the other, similar species of the *C. saundersi* complex. We examined the three syntype minor workers of *C. badia* in the Oxford University Museum of Natural History. To fix the identity of this taxon, we have chosen the syntype from Singapore (imaged by AntWeb.org under CASENT0901897) as the lectotype of *Formica badia*. The two syntypes from Sarawak are in a relatively poor condition, which does not allow a complete morphometric analysis, and therefore the conspecificity with the *C. badia* type remains doubtful. We were not successful in obtaining fresh material of *C. badia* from Singapore, but a nest sample (minors only) from southern Thailand (Trang Province) which agrees well with the lectotype in morphology, especially morphometry, was available for a molecular analysis. It shows that *C. badia* and *C. explodens* sp. n. are closely related, but distinct (Fig. 1). Although very similar to *C. explodens* sp. n. in overall habitus and colouration, the examined *C. badia* minor workers are on average somewhat larger with less size variation (HW 1.22–1.57 vs. 1.46–1.59) and possess longer appendages (e.g., FeI 123–151 vs. 141–168; see Fig. 8a).

We examined two syntype minor workers of *Colobopsis saundersi* from Myanmar (“Tenasserim, Thagata”, one illustrated by AntWeb.org under CASENT0905463). Morphometric analysis revealed no differences between the types of *C. saundersi* and *C. badia*, suggesting that the two species should be synonymized. *Colobopsis saundersi* was considered a junior synonym of *C. badia* by Carlo Emery himself (Emery 1896) but revived from synonymy by Bingham (1903) without providing a reason. The large geographic distance of the type locality of *C. saundersi* and some minor differences in morphology led us to the decision to refrain from a formal synonymization at this time. A comparative molecular analysis of specimens from the type localities (Myanmar, Singapore) would most likely be necessary to corroborate this synonymy.

### Morphology of males

The morphology of males of the tribe Camponotini is insufficiently studied, so that a complete comparison at generic level is not possible. The modified (enlarged) first funicular segment is presumably characteristic for males of *Colobopsis*. This characteristic has been described in the type species, *Colobopsis truncata* (Spinola, 1808), by Kutter (1977) and has been equally observed in several species of the *C. cylindrica* group.

Males of the COCY group have previously been described for three species (see below). However, these descriptions largely lack the necessary details to meaningfully compare taxa. No previous accounts of genital morphology or illustrations of male specimens have been found in the literature.

*Colobopsis badia*: Viehmeyer (1916) gives a brief description of a male from Singapore. Colouration, size, proportions of head and ocelli, as well as the enlarged first funicular segment correspond well to the examined male from Thailand.

*Colobopsis severini* (Forel, 1909): The extremely brief description of a male from the island Labuan (near Borneo) is not sufficient to draw any meaningful taxonomic conclusions.

*Colobopsis leonardi* (Emery, 1889): Karawajew (1929) gives a rather detailed description of males collected within a nest-series on Sumatra. The correct species identification by Karawajew is uncertain; the series may belong to another species of the *C. leonardi* complex as well. The pattern of pilosity on the gaster, with standing setae only present on the posterior half, also corresponds to our observations in males of the *C. saundersi* complex.

According to our knowledge, males of the *C. cylindrica* group can be distinguished from other Southeast Asian *Colobopsis* species by the relatively rich subdecumbent pilosity and the dense microreticulation of gastral tergites.

### Biology

The behavioural observations on *C. explodens* sp. n. conducted at KBFSC revealed multiple modes of behaviour that are either poorly studied or new to science.

The diurnal activity pattern, as well as the positive influence of high temperatures correspond to the results of previous studies in related taxa (see Hamdan et al. 2013). Similarly large colonies containing several thousand individuals and extending to multiple trees and / or artificial nesting structures have also been described for other members of the genus (Federle et al. 1998, Laciny et al. 2017). However, it is still unclear whether individual workers are linked to certain parts of the colony or whether all foragers can move freely through the entire territory of the colony. An interesting and hitherto undescribed phenomenon in this regard is the presence of one or multiple “guards” at the artificial nest’s entrance: These minor workers were frequently observed to touch any incoming or leaving workers with their antennae. In some instances, returning foragers were delayed or altogether denied entrance by the guarding ants. One reason for this may be that some workers are linked to different parts of the colony. Alternatively, the observed guarding behaviour may be related to the limited capacity of the artificial nest, which is also suggested by the conspicuously balanced numbers of workers entering and leaving the nest during times of foraging activity. These behavioural patterns are hitherto undescribed and must be investigated in future studies.

A further noteworthy activity observed in foraging workers was so-called “grazing” behaviour, in which minor workers were frequently seen using their mandibles to pluck and chew at various mosses, lichens, and other epiphytes on the bark of trees or other surfaces. While this activity can last for up to one hour at a time, its exact purpose remains unclear. It is possible that minor workers cut and consume parts of the plants and microorganisms or merely ingest fluids. As previous analyses of nitrogen isotopes (Davidson et al. 2016) suggest a largely plant-based diet for COCY ants, it seems likely that “grazing” contributes to their nutrition. However, other previously hypothesized modes of nutrition, such as tending of scale insects (Davidson et al. 2016) were not observed, and recent investigations on *Colobopsis leonardi* (Emery, 1889) (Zettel et al., ms submitted to Asian Myrmecology) even suggest a higher prevalence of carnivory in COCY ants than previously suspected.

## Supplementary Material

XML Treatment for Colobopsis explodens

XML Treatment for Colobopsis badia

## Supplementary materials

Supplementary material 1 Figure S1. Living workers of *C. explodens* sp. n. on a detached branch containing a nest fragment Data type: multimedia Explanation note: **a** Minor worker with characteristically raised gaster **b** Major worker with phragmotic head to close nest entrances. Photo: H. Wiesbauer This is an open access article distributed under the terms of the Creative Commons Attribution License (CC BY 4.0), which permits unrestricted use, distribution, and reproduction in any medium, provided the original author and source are credited.Laciny A, Zettel H, Kopchinskiy A, Pretzer C, Pal A, Salim KA, Rahimi MJ, Hoenigsberger M, Lim L, Jaitrong W, Druzhinina IS

Supplementary material 2 Figure S2. Natural nest of *C. explodens* sp. n. in a dead branch in the high canopy of *S. johorensis* (the main host tree for the model colony) Data type: multimedia Explanation note: **a** Camera setup in the canopy, white arrow marks the nest entrance **b, c** Entrance and minor workers. This is an open access article distributed under the terms of the Creative Commons Attribution License (CC BY 4.0), which permits unrestricted use, distribution, and reproduction in any medium, provided the original author and source are credited.Laciny A, Zettel H, Kopchinskiy A, Pretzer C, Pal A, Salim KA, Rahimi MJ, Hoenigsberger M, Lim L, Jaitrong W, Druzhinina IS

Supplementary material 3 Figure S3. Interior of the natural nest of *C. explodens* sp. n. found in a dead tree branch of *S. johorensis* on the forest floor Data type: multimedia Explanation note: **a** Longitudinal section of the nest **b** Nest cavity. Sawdust is an artefact due to cutting. **c** Enlarged view of the nest cavity with a chamber made of dark carton **d** Dealate gyne and eggs found inside the chamber shown on **c**. The nest contained at least three such chambers. This is an open access article distributed under the terms of the Creative Commons Attribution License (CC BY 4.0), which permits unrestricted use, distribution, and reproduction in any medium, provided the original author and source are credited.Laciny A, Zettel H, Kopchinskiy A, Pretzer C, Pal A, Salim KA, Rahimi MJ, Hoenigsberger M, Lim L, Jaitrong W, Druzhinina IS

Supplementary material 4 Figure S4. Autothysis as defensive behaviour in an experimental setting Data type: multimedia Explanation note: A worker of the predatory species *Oecophylla smaragdina* is attacked by three minor workers of *C. explodens* sp. n.; all four animals died after the encounter. Black arrows indicate the yellow secretion expelled by rupture of the mandibular gland reservoirs. This is an open access article distributed under the terms of the Creative Commons Attribution License (CC BY 4.0), which permits unrestricted use, distribution, and reproduction in any medium, provided the original author and source are credited.Laciny A, Zettel H, Kopchinskiy A, Pretzer C, Pal A, Salim KA, Rahimi MJ, Hoenigsberger M, Lim L, Jaitrong W, Druzhinina IS

Supplementary material 5 Figure S5. Pupa of *C. explodens* sp. n. found inside the opened natural nest Data type: multimedia Explanation note: Note the absence of a pupal cocoon which is diagnostic for *Colobopsis*. This is an open access article distributed under the terms of the Creative Commons Attribution License (CC BY 4.0), which permits unrestricted use, distribution, and reproduction in any medium, provided the original author and source are credited.Laciny A, Zettel H, Kopchinskiy A, Pretzer C, Pal A, Salim KA, Rahimi MJ, Hoenigsberger M, Lim L, Jaitrong W, Druzhinina IS

Supplementary material 6 Table S6. Data type: specimens data Explanation note: “measurements”: Complete dataset of measurements, indices, locality data and type status of all measured specimens of *C. explodens* sp. n. “activity”: Activity of minor workers of *C. explodens* sp. n. at the entrance of the artificial nest #38. “accession numbers”: Complete list of accession numbers at NCBI GenBank for all organisms treated in this study. This is an open access article distributed under the terms of the Creative Commons Attribution License (CC BY 4.0), which permits unrestricted use, distribution, and reproduction in any medium, provided the original author and source are credited.Laciny A, Zettel H, Kopchinskiy A, Pretzer C, Pal A, Salim KA, Rahimi MJ, Hoenigsberger M, Lim L, Jaitrong W, Druzhinina IS

Supplementary material 7 Video S7. Video depicting habitat, nesting sites and behaviour of *C. explodens* sp. n. Data type: multimedia Explanation note: Video depicting habitat, nesting sites and behaviour of *C. explodens* sp. n. accessible under http://explodingants.com/index.php/publications/colobopsis-explodens This is an open access article distributed under the terms of the Creative Commons Attribution License (CC BY 4.0), which permits unrestricted use, distribution, and reproduction in any medium, provided the original author and source are credited.Laciny A, Zettel H, Kopchinskiy A, Pretzer C, Pal A, Salim KA, Rahimi MJ, Hoenigsberger M, Lim L, Jaitrong W, Druzhinina IS
