# Supplementary figures and images for: Colobopsis explodens sp. n., model species for studies on “exploding ants” (Hymenoptera, Formicidae), with biological notes and first illustrations of males of the Colobopsis cylindrica group
Source: Zookeys. 2018 Apr 19;(751):1–40. doi: 10.3897/zookeys.751.22661 (PMC5919914; doi:10.3897/zookeys.751.22661)

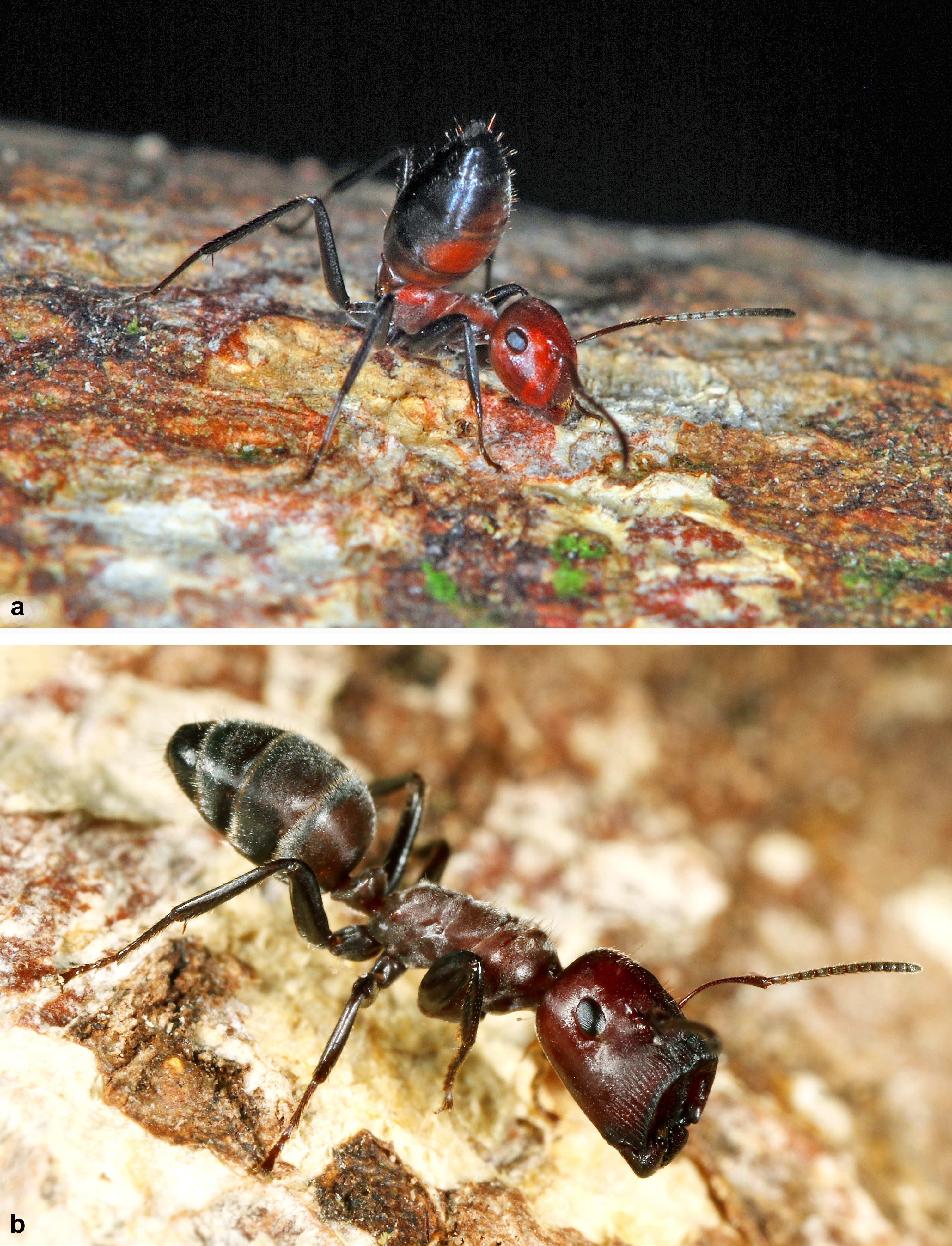

Supplement: Supplementary material 1 — Figure S1. Living workers of C. explodens sp. n. on a detached branch containing a nest fragment [file zookeys-751-001-s001.jpg]

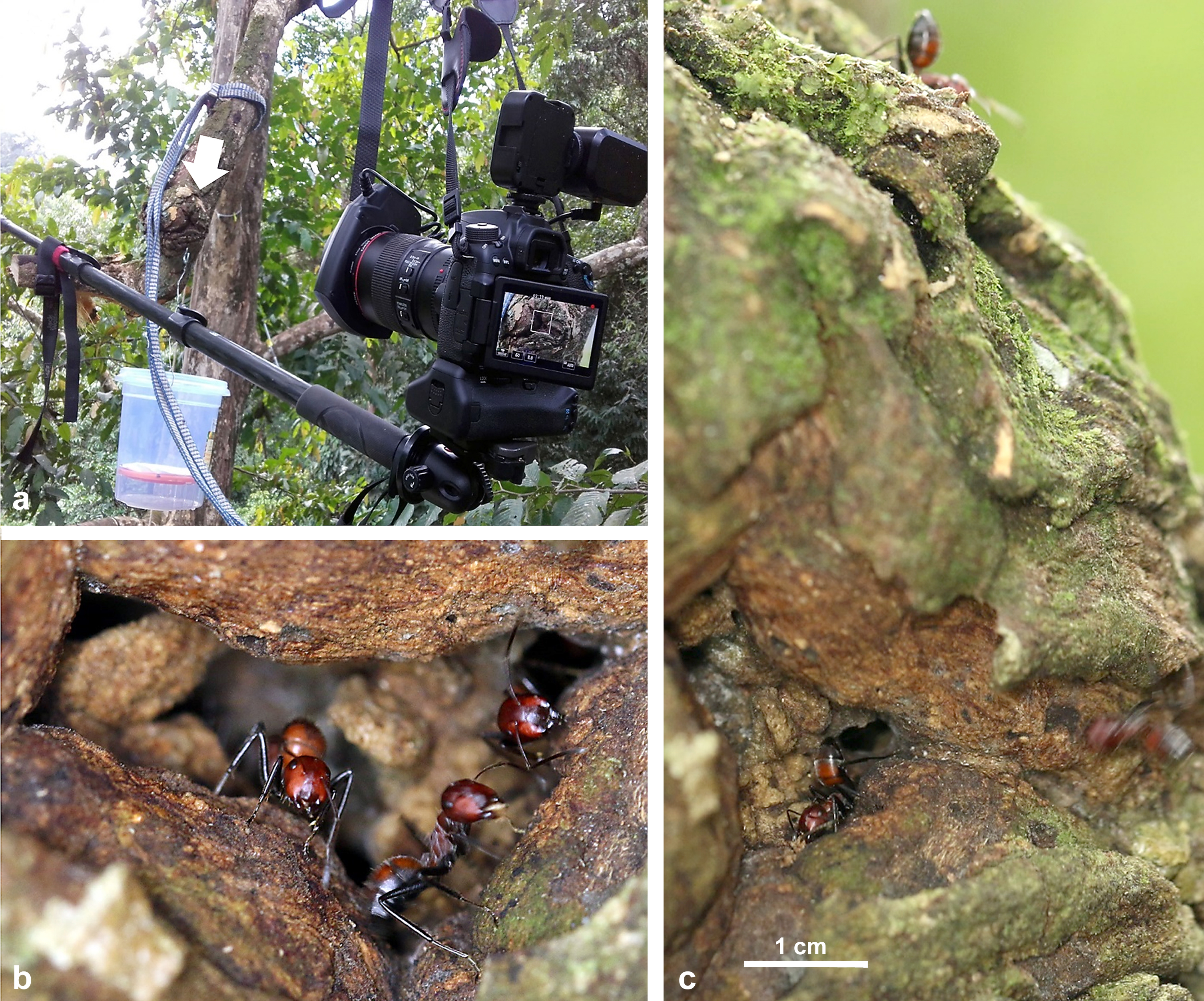

Supplement: Supplementary material 2 — Figure S2. Natural nest of C. explodens sp. n. in a dead branch in the high canopy of S. johorensis (the main host tree for the model colony) [file zookeys-751-001-s002.jpg]

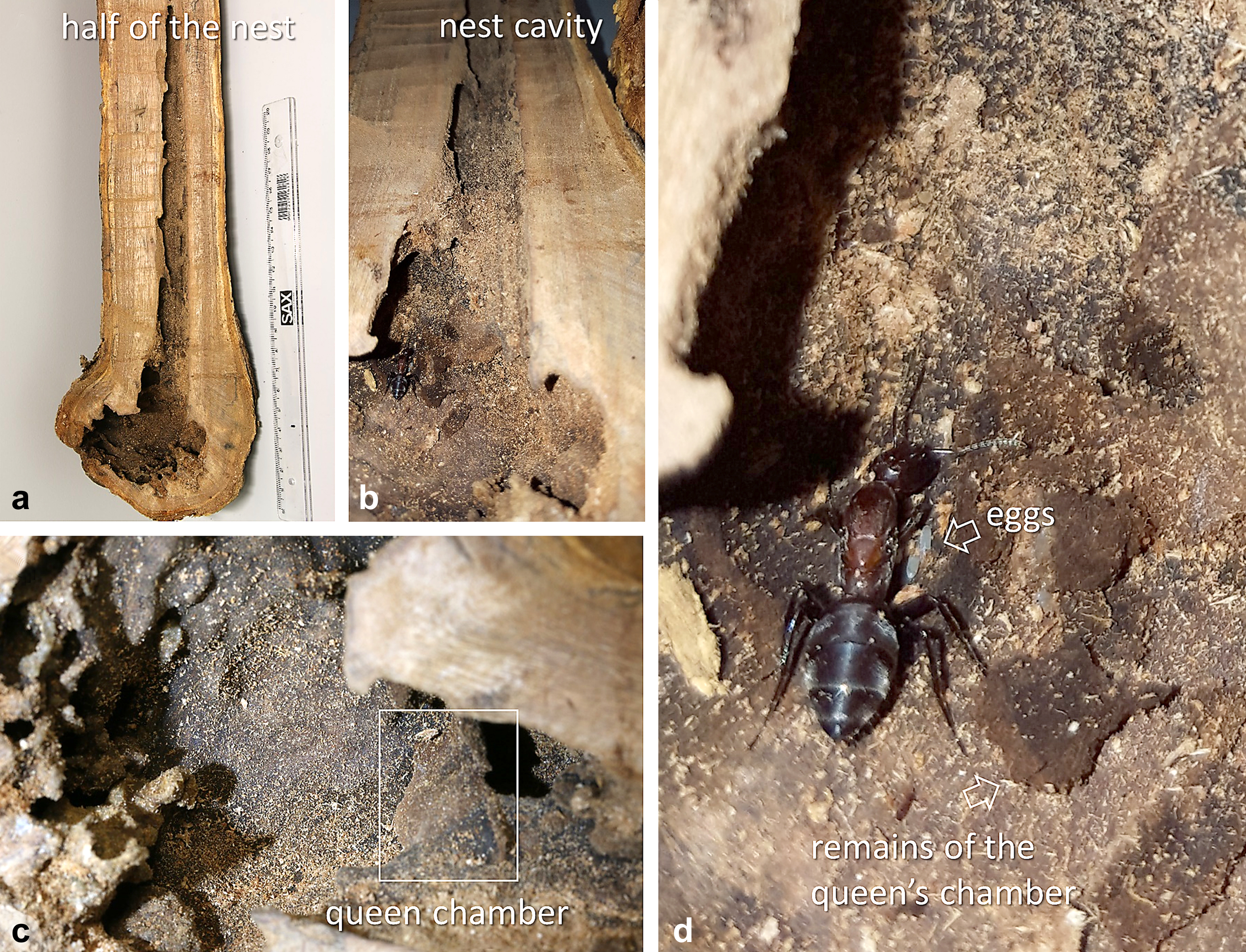

Supplement: Supplementary material 3 — Figure S3. Interior of the natural nest of C. explodens sp. n. found in a dead tree branch of S. johorensis on the forest floor [file zookeys-751-001-s003.jpg]

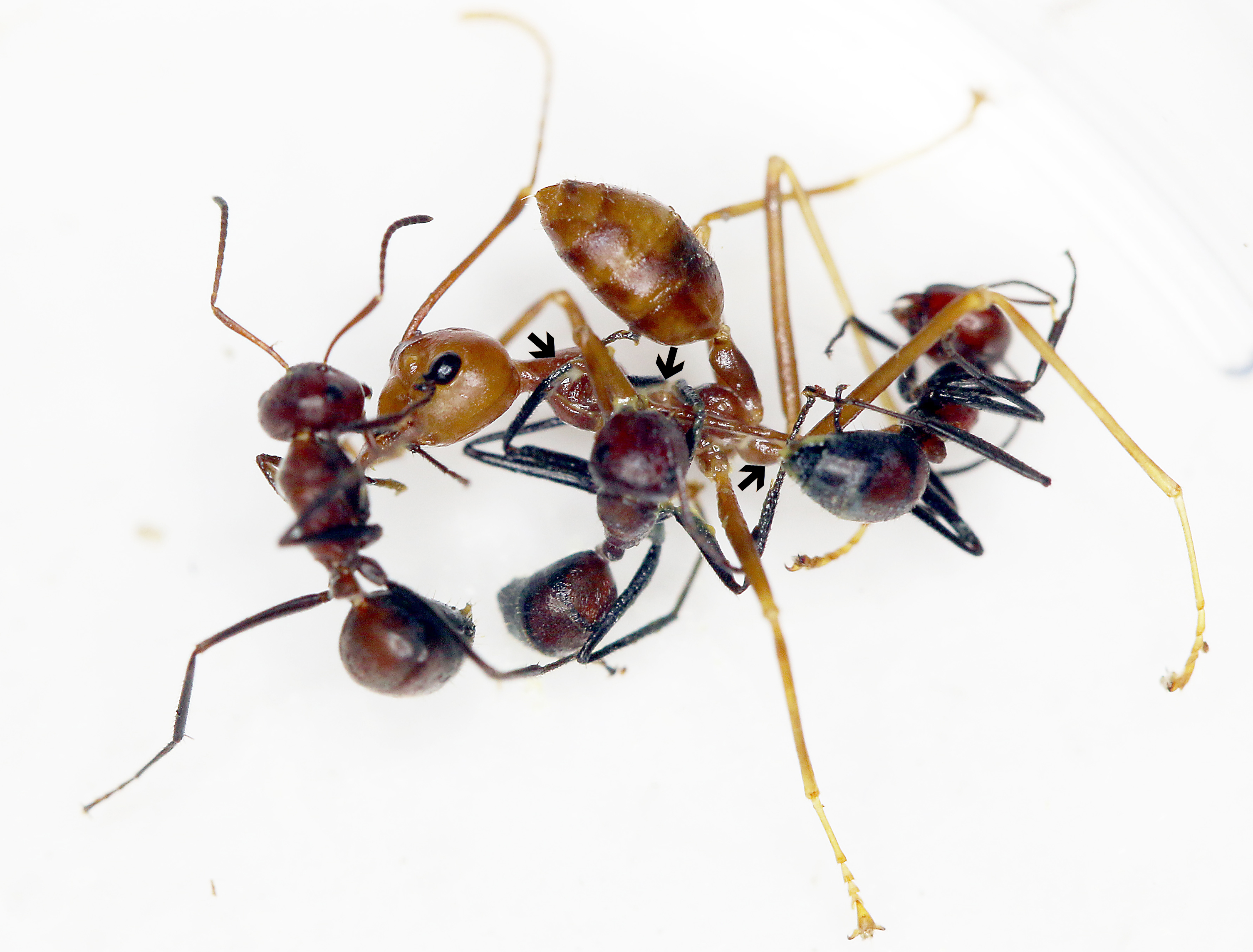

Supplement: Supplementary material 4 — Figure S4. Autothysis as defensive behaviour in an experimental setting [file zookeys-751-001-s004.jpg]

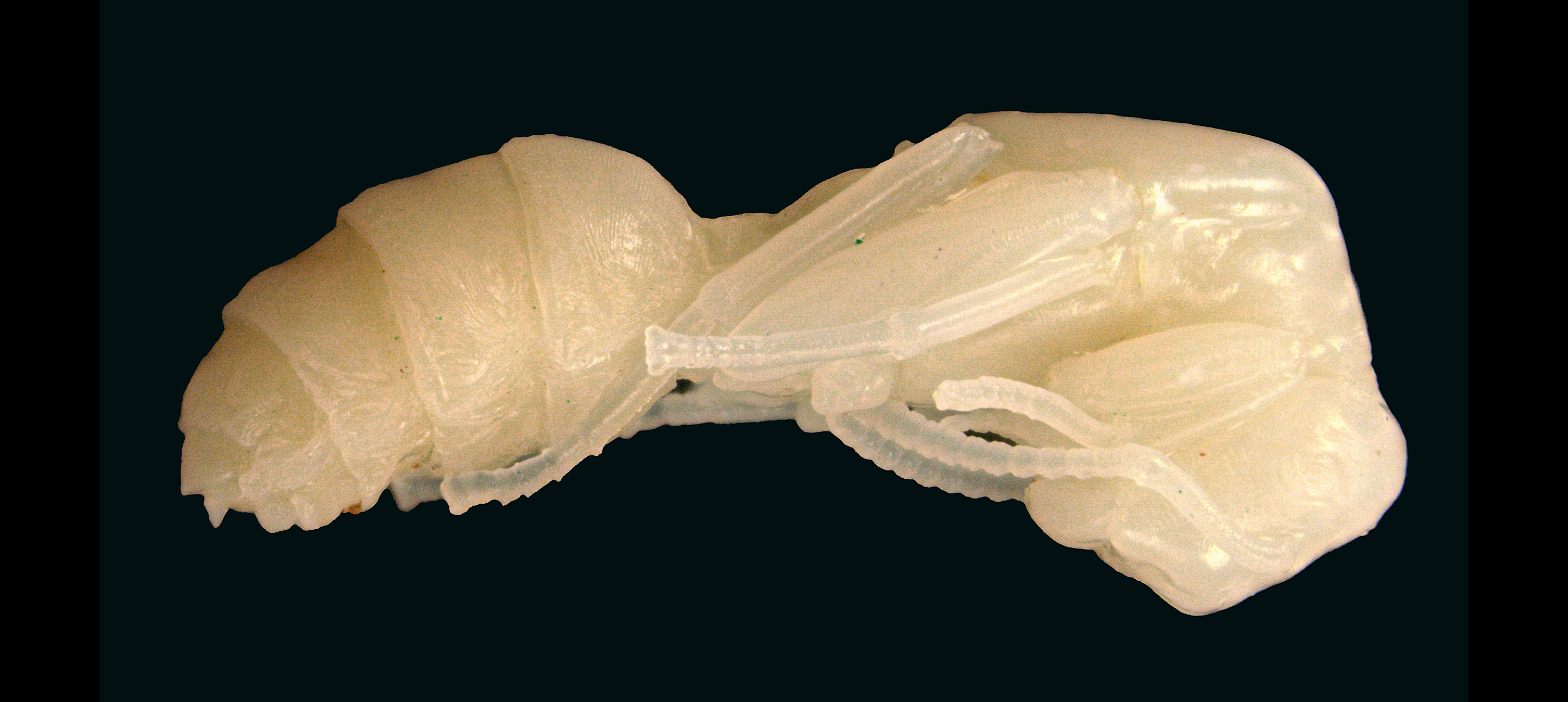

Supplement: Supplementary material 5 — Figure S5. Pupa of C. explodens sp. n. found inside the opened natural nest [file zookeys-751-001-s005.jpg]
